# In the Absence of Effector Proteins, the *Pseudomonas aeruginosa* Type Three Secretion System Needle Tip Complex Contributes to Lung Injury and Systemic Inflammatory Responses

**DOI:** 10.1371/journal.pone.0081792

**Published:** 2013-11-27

**Authors:** Jonathon P. Audia, Ashley S. Lindsey, Nicole A. Housley, Courtney R. Ochoa, Chun Zhou, Michie Toba, Masahiko Oka, Naga S. Annamdevula, Meshann S. Fitzgerald, Dara W. Frank, Diego F. Alvarez

**Affiliations:** 1 Department of Microbiology and Immunology, University of South Alabama, Mobile, Alabama, United States of America; 2 Department of Internal Medicine, University of South Alabama, Mobile, Alabama, United States of America; 3 Department of Pharmacology, University of South Alabama, Mobile, Alabama, United States of America; 4 Center for Lung Biology, University of South Alabama, Mobile, Alabama, United States of America; 5 Department of Chemical and Biomolecular Engineering, University of South Alabama, Mobile, Alabama, United States of America; 6 Department of Microbiology and Molecular Genetics, Medical College of Wisconsin, Milwaukee, Wisconsin, United States of America; UC Berkeley, United States of America

## Abstract

Herein we describe a pathogenic role for the *Pseudomonas aeruginosa* type three secretion system (T3SS) needle tip complex protein, PcrV, in causing lung endothelial injury. We first established a model in which *P. aeruginosa* wild type strain PA103 caused pneumonia-induced sepsis and distal organ dysfunction. Interestingly, a PA103 derivative strain lacking its two known secreted effectors, ExoU and ExoT [denoted PA103 (ΔU/ΔT)], also caused sepsis and modest distal organ injury whereas an isogenic PA103 strain lacking the T3SS needle tip complex assembly protein [denoted PA103 (ΔPcrV)] did not. PA103 (ΔU/ΔT) infection caused neutrophil influx into the lung parenchyma, lung endothelial injury, and distal organ injury (reminiscent of sepsis). In contrast, PA103 (ΔPcrV) infection caused nominal neutrophil infiltration and lung endothelial injury, but no distal organ injury. We further examined pathogenic mechanisms of the T3SS needle tip complex using cultured rat pulmonary microvascular endothelial cells (PMVECs) and revealed a two-phase, temporal nature of infection. At 5-hours post-inoculation (early phase infection), PA103 (ΔU/ΔT) elicited PMVEC barrier disruption via perturbation of the actin cytoskeleton and did so in a cell death-independent manner. Conversely, PA103 (ΔPcrV) infection did not elicit early phase PMVEC barrier disruption. At 24-hours post-inoculation (late phase infection), PA103 (ΔU/ΔT) induced PMVEC damage and death that displayed an apoptotic component. Although PA103 (ΔPcrV) infection induced late phase PMVEC damage and death, it did so to an attenuated extent. The PA103 (ΔU/ΔT) and PA103 (ΔPcrV) mutants grew at similar rates and were able to adhere equally to PMVECs post-inoculation indicating that the observed differences in damage and barrier disruption are likely attributable to T3SS needle tip complex-mediated pathogenic differences post host cell attachment. Together, these infection data suggest that the T3SS needle tip complex and/or another undefined secreted effector(s) are important determinants of *P. aeruginosa* pneumonia-induced lung endothelial barrier disruption.

## Introduction

*Pseudomonas aeruginosa* is a Gram-negative, opportunistic pathogen that causes nosocomial infections in patients undergoing mechanical ventilation and in people that are immunocompromised (e.g., severe burn) [1–6]. This pathogen is also a major cause of chronic infections in cystic fibrosis patients leading to increased mortality [7–10]. *P. aeruginosa* is a ubiquitous environmental microbe and is typically considered an extracellular pathogen that attaches to eukaryotic cells and/or forms biofilms to establish host colonization [11–13]. Cellular invasive *P. aeruginosa* phenotypes have been described [14–16] but the role of intracellular pseudomonads in pathogenesis remains unclear. In susceptible hosts, acute and chronic *P. aeruginosa* infections are difficult to treat owing to endogenous antibiotic resistance systems such as multi-drug efflux pumps and biofilms.

*P. aeruginosa* is a leading cause of pneumonia-induced Acute Respiratory Distress Syndrome (ARDS) [1,3,4,8,9,17,18]. Upon infection of the airway, pseudomonads infect alveolar epithelial cells and resident macrophages, eliciting release of pro-inflammatory cytokines to recruit immune cells into the lung parenchyma and airspaces [17,19–22]. Subsequent damage to the alveolar epithelial barrier allows direct infection of lung endothelial cells that, along with the deleterious effects of endotoxin and cytokines, precipitate vascular endothelial barrier disruption [2,20,23–27].

Pulmonary microvascular endothelial cells (PMVECs) form contiguous, semi-permeable barriers between the bloodstream and the interstitial space, limiting the vectorial movement of fluid, solute, macromolecules, and gases [28–35]. Thus, disruption of PMVEC barriers by *P. aeruginosa* infection results in the hallmark features of ARDS, namely, increased neutrophil infiltration, increased fluid filtration, pulmonary edema, and low blood oxygen levels [36–38].

The propensity for *P. aeruginosa* infection to elicit ARDS and the attendant PMVEC injury is largely dependent on the cadre of virulence factors available to the pathogen. In particular, highly virulent clinical *P. aeruginosa* isolates cause cellular damage through the use of a type three secretion system (T3SS) that injects effector proteins directly into the cytoplasm of an infected eukaryotic cell [4,10,39–42]. To date, four *P. aeruginosa* T3SS-delivered effector proteins (ExoU, ExoS, ExoT, and ExoY) have been described [40,43]. All of these T3SS-delivered effector proteins are notoriously dependent upon eukaryotic co-factors to activate their enzymatic activities. ExoU is a potent phospholipase A_2_ cytotoxin that rapidly causes eukaryotic cell lysis and stimulates lipid signal transduction cascades [44,45]. ExoU activation is mediated by interactions with eukaryotic mono- and poly-ubiquitin, and ubiquitinylated proteins such as Cu/Zn superoxide dismutase 1 [46–49]. ExoS and ExoT are dual functioning Rho GTPase activating and ADP-ribosyltranferase effectors that disrupt eukaryotic cell signaling, prevent phagocytosis, and mediate the pathogen’s ability to disrupt the epithelial barrier [43,45,50,51]. ExoS and ExoT are activated by the 14-3-3 family of proteins. ExoY is an adenylyl cyclase that increases levels of cAMP in the cytoplasm disrupting PMVEC barrier function [41,43]. A eukaryotic co-factor for ExoY has yet to be identified. Interestingly, all four effector proteins are rarely found together in a given clinical isolate with ExoU and ExoS being almost mutually exclusive. Of the four effector proteins, ExoU and ExoS are known to elicit cellular cytotoxicity, and strains that secrete ExoU are associated with poor patient outcome [4,39,52]. It is thought that the predominant role of the effector proteins in pathogenesis is to mediate cellular damage, impair phagocyte engulfment, and suppress immune cell infiltration, which ultimately facilitate bacterial persistence in the lung and subsequent dissemination [4,21,44,45,51–53].

The T3SS is analogized as a highly complex and ordered molecular syringe that directly facilitates energy-dependent intoxication of eukaryotic host cell cytoplasm with the effector proteins [54–56]. In the bacterial cytoplasm, the activity of molecular chaperones and ATPases mobilize the effector proteins for secretion [54]. At the eukaryotic cell surface the P. *aeruginosa* PopB, PopD, and PcrV proteins constitute the T3SS needle tip complex; forming a membrane pore through which the effector proteins translocate into host cells [7,57,58]. The pore-forming potential of the PopBD/PcrV complex has been demonstrated using red blood cell hemolysis assays [58–61]. Regulation of membrane pore formation by the T3SS needle tip complex is dependent upon PcrV. Indeed, *pcrV* null mutant strains of *P. aeruginosa* are unable to directly inject effector proteins into eukaryotic host cells but instead, display dysregulated secretion of effector proteins into the culture medium [62,63]. As a result, *pcrV* mutants are avirulent and the PcrV protein is being developed and tested as a protective antigen against *P. aeruginosa*-induced pneumonia.

Beyond its known requirement for regulated effector protein delivery, the T3SS needle tip complex itself is emerging as a virulence factor that mediates critical pathogen-host interactions [36,62]. In *P. aeruginosa* strains that do not encode any of the four known effector proteins (*ΔexoU/S/T/Y*), the T3SS needle tip complex alone has been shown to mediate virulence-associated effects such as pro-inflammatory cytokine induction, immune cell infiltration into the lung, immune cell death, and mortality in animal models of infection [36]. Indeed, clinical isolates that express only a functional T3SS with no effector proteins have been isolated from patients and correlated with increased morbidity and mortality compared to strains lacking a functional T3SS [4]. However, no studies to date have directly assessed a role for the P. *aeruginosa* T3SS needle tip complex in non-immune cell mediated pathogenesis of lung injury and PMVEC barrier dysfunction during pneumonia-induced ARDS. To this end, we have developed rat airway- and isolated PMVEC-infection model systems and described a role for the P. *aeruginosa* T3SS needle tip complex protein, PcrV, as a virulence factor that mediates PMVEC barrier disruption, lung injury, sepsis, and systemic dysfunction.

## Results

### *P. aeruginosa* strain PA103 Causes Sepsis and Multi-Organ Dysfunction in a Rat Airway Infection Model

**Figure 1:**
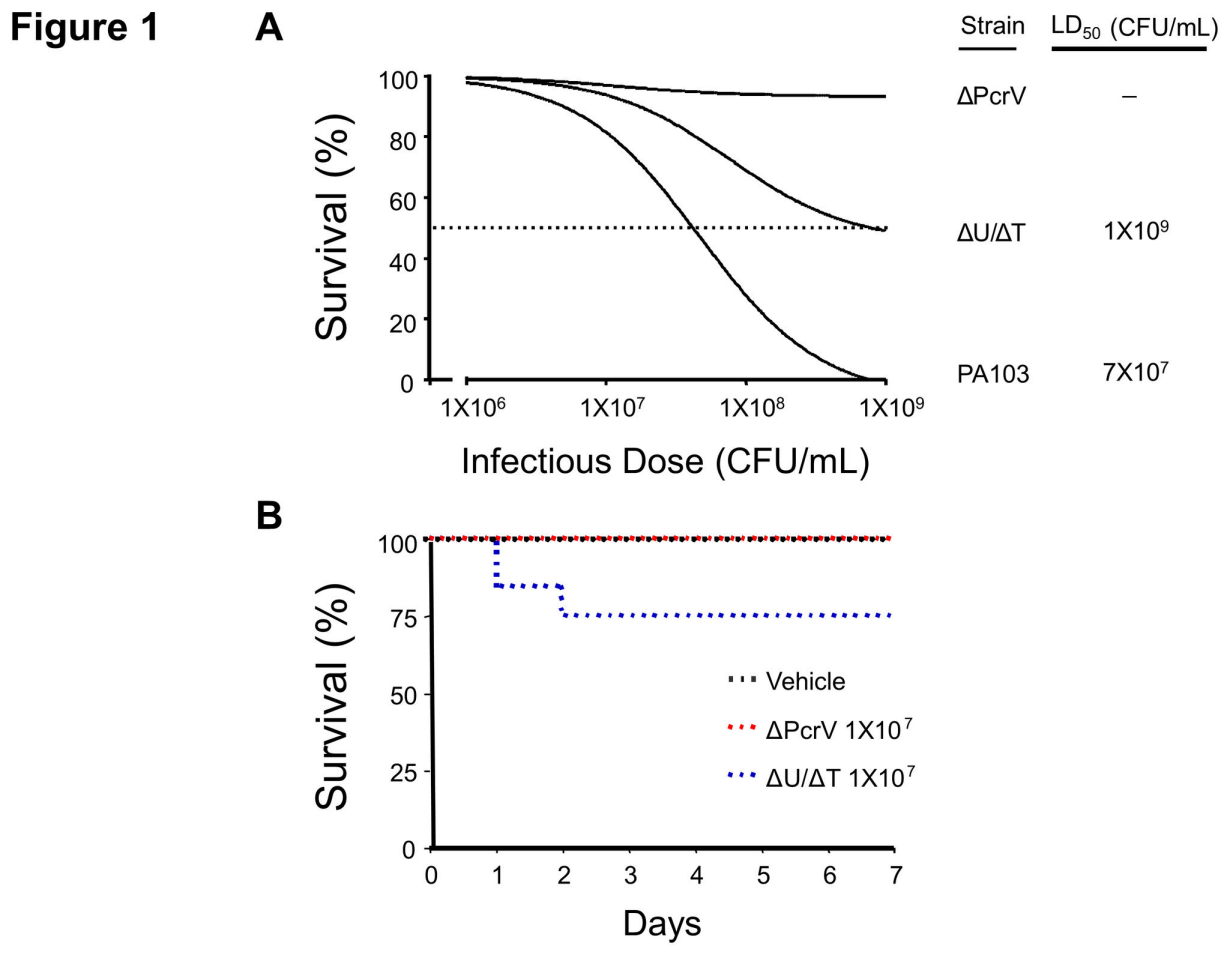
*P. aeruginosa* T3SS needle tip complex protein PcrV associates with mortality in a rat model of lung infection. **A**. Intratracheal instillation of the P. *aeruginosa* wild type PA103 and mutant PA103 (ΔU/ΔT) strains into rats resulted in dose-dependent mortality compared to the mutant PA103 (ΔPcrV) which caused no mortality at 1-week post-infection. Doses (CFUs/mL) are listed in the figure. Data represent at least n=20 per group. **B**. One week survival analysis (Kaplan Meier curve) at a sub-LD_50_ dose (~1 x 10^7^ CFUs). Mortality in the PA103 (ΔU/ΔT) cohort was observed within 48-hours post-infection. Black dotted line represents vehicle control (saline solution), red dotted line represents infection with PA103 (ΔPcrV), and blue dotted line represents Infection with PA103 (ΔU/ΔT). Data represent at least n=20 per group.

*P. aeruginosa* strain PA103 is a virulent clinical isolate that encodes only two T3SS effector proteins, namely ExoU and ExoT. We used strain PA103 to establish an airway infection model where lung injury is induced by intratracheal instillation of bacteria into adult, male CD rats. Figure **1A** shows the one-week mortality dose-response curve from rats inoculated with PA103 (wild type) where ~7 x 10^7^ colony forming units (CFUs) resulted in a 50% mortality rate. In patients, airway infection with *P. aeruginosa* often progresses to sepsis and, in some instances, multiple organ dysfunction. Table **1** shows animal biometrics at 48-hours post-inoculation with a moderate dose of PA103 (~2.5 x 10^7^ CFUs). Infection elicited elevated body temperature and adversely affected liver function as indicated by elevated levels of AST (Table **1**), and liver pathological analysis revealed the presence of periportal inflammation (Figure **S1**). PA103 infection also resulted in depressed cardiac function as indicated by decreased dP/dt and stroke work (Table **1**), and bacteria were detected in the blood and lungs by direct plating on *Pseudomonas* isolation agar (data not shown). PA103 infection increased creatinine levels when compared to vehicle control (Table **1**), and kidney pathological analysis revealed the presence of tubular casts indicative of sepsis-induced injury (Figure **S2**). In addition, PA103 infection caused a systemic neutrophilia, accompanied by a marked lymphopenia (Table **1**). Together these data suggest that our rat airway instillation model using PA103 recapitulates infection-induced morbidity, sepsis, multi-organ dysfunction, and mortality.

**Table 1:** Biometrics of Vehicle Control and *P. aeruginosa*-Infected Animals ^***a***^.

	**Vehicle**	**PA103**	**ΔPcrV**	**ΔU/ΔT**	***P* value**
Temperature (rectal, °C)	37.1 ± 0.1	38.8 ± 0.3 **^*c*^**	37.3 ± 0.2	38.2 ± 0.3 **^*c*^**	0.0069 **^*b*^**
Total leukocytes (counts/µL)	5293 ± 592	6497 ± 516	6530 ± 35	4433 ± 371	0.0540 **^*b*^**
Neutrophil differential (%)	11 ± 1	86 ± 2 **^*d*^**	33 ± 11 **^*d*^**	65 ± 4 **^*d*^**	0.0001 **^*b*^**
Lymphocyte differential (%)	88 ± 1	14 ± 2 **^*d*^**	66 ± 10 **^*d*^**	34 ± 6 **^*d*^**	0.0001 **^*b*^**
AST (U/L)	71 ± 2	127 ± 21 **^*e*^**	89 ± 6	109 ± 6 **^*e*^**	0.0069 **^*b*^**
Creatinine (mg/dL)	0.5 ± 0.0	0.7 ± 0.1 **^*f*^**	0.6 ± 0.1	0.7 ± 0.1 **^*f*^**	0.0720 **^*b*^**
dP/dt (mmHg/sec)	13438 ± 2235	6687 ± 399 **^*g*^**	N.D.	N.D.	0.0410 **^*g*^**
Stroke work (mmHg x mL)	28 ± 3	13 ± 1 **^*g*^**	N.D.	N.D.	0.0066 **^*g*^**

^***a***^ Anesthetized adult male CD rats were intratracheally instilled with saline solution (vehicle) or individually with the three different *P. aeruginosa* strains at ~2.5 x 10^7^ CFU/mL. At 48-h post-infection, animals were anesthetized and body temperature measured rectally. Blood chemistry analysis was used to assess liver function (aspartate aminotransferase, AST) and kidney function (creatinine). Left ventricular function was assessed by two contractility indexes, namely dP/dt and stroke work.

^***b***^ Comparisons among groups were made using a one-way ANOVA and P < 0.05 was considered statistically significant. Significant differences among groups were established by Newman-Keuls *post-hoc* analysis.

^***c***^ Significantly different compared to vehicle and ΔPcrV.

^***d***^ Significantly different among all groups.

^***e***^ Significantly different compared to vehicle.

^***f***^ Not significantly different by one-way ANOVA but significantly different when individually compared to vehicle using an unpaired *t*-test (*P* < 0.05).

^***g***^ Significantly different compared to vehicle using an unpaired *t*-test (*P* < 0.05).

N.D., not determined.

### The P. *aeruginosa* PcrV Protein Contributes to Mortality and Morbidity in a Rat Airway Infection Model

To examine the role of the T3SS needle tip complex (specifically, PcrV) in *P. aeruginosa* virulence, we compared rats infected with an isogenic PA103 mutant encoding an intact T3SS needle tip complex but defective in the synthesis of ExoU and ExoT genes [PA103 (ΔU/ΔT)] to rats infected with an isogenic PA103 mutant deleted for the *pcrV* gene [PA103 (ΔPcrV)]. Figure **1A** shows that inoculation with ~1 x 10^9^ CFUs of the PA103 (ΔU/ΔT) mutant resulted in a 50% mortality rate at one week post-inoculation whereas the PA103 (ΔPcrV) mutant did not cause mortality at the maximal dose tested. Thus, although the PA103 (ΔU/ΔT) mutant is attenuated compared to wild type PA103, it causes infection-induced mortality in a PcrV-dependent manner.

While it is clear that morbidity in *P. aeruginosa*-infected patients strongly correlates with secretion of effector proteins [4], it is poorly understood whether *P. aeruginosa* expressing only a function T3SS needle tip complex elicits lung endothelial injury via immune cell-independent mechanisms [36]. Figure **1B** shows a Kaplan-Meier curve at an inoculation dose of ~1X10^7^ CFUs, where ~75% and 100% of the animals survive at 48-hours post-inoculation with PA103 (ΔU/ΔT) or PA103 (ΔPcrV), respectively. Once established for the two mutant *P. aeruginosa* strains, this moderate infectious dose was used in all subsequent *in vivo* experiments to assess indexes of injury at 48-hours post-inoculation. PA103 (ΔU/ΔT) elicited elevated body temperature and adversely affected liver function as indicated by elevated AST (Table **1**), and the presence of periportal inflammation, whereas PA103 (ΔPcrV) did not (Figure **S1**). Only PA103 (ΔU/ΔT) could be detected in the blood by direct plating (data not shown). While neither *P. aeruginosa* mutant strain affected kidney function as measured by creatinine levels (Table **1**), infection with PA103 (ΔU/ΔT) caused modest formation of tubular casts, whereas PA103 (ΔPcrV) did not (Figure **S2**). Finally, infection with PA103 (ΔU/ΔT) also caused a systemic leukopenia with neutrophilia, accompanied by a marked lymphopenia (Table **1**). Together these data suggest that PcrV and the T3SS needle tip complex contribute to infection-induced morbidity including sepsis- and multi-organ dysfunction.

### The P. *aeruginosa* PcrV Protein Contributes to Lung Injury and Vascular Endothelial Barrier Disruption in a Rat Airway Infection Model

**Figure 2:**
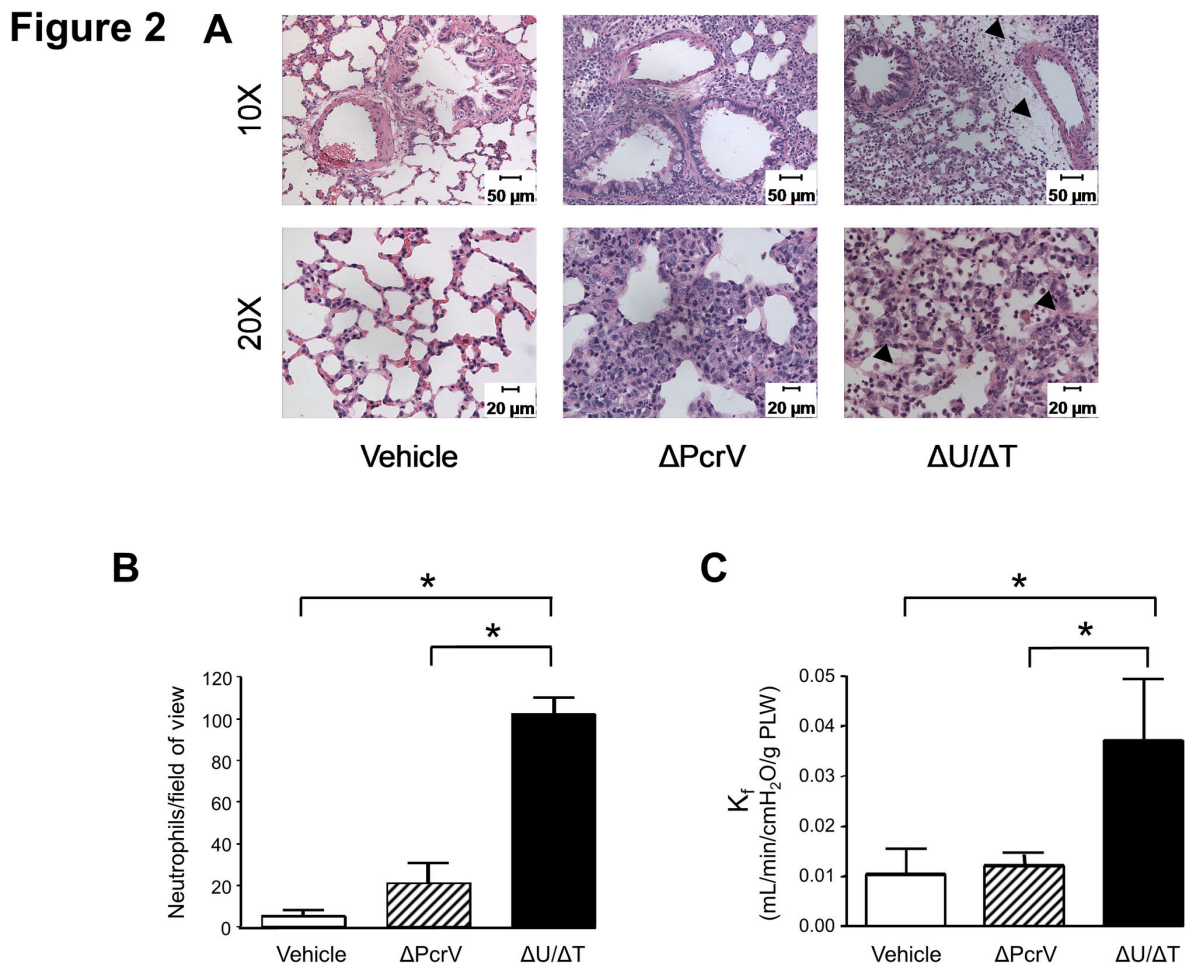
*P. aeruginosa* T3SS needle tip complex protein PcrV associates with lung injury. **A**. Injured areas of the lung were prepared for histological hematoxylin/eosin analysis at 48-hours post-inoculation. Animals were inoculated with saline solution (vehicle control), with ~1 x 10^7^ CFUs of PA103 (ΔU/ΔT), or with ~1 x 10^7^ CFUs of PA103 (ΔPcrV). Lung sections imaged at 10X (top panels) and 20X (bottom panels) revealed that intratracheal instillation of PA103 (ΔU/ΔT) or PA103 (ΔPcrV) caused lung injury. Inoculation with PA103 (ΔU/ΔT) resulted in fluid accumulation around the bronchovascular bundles (vascular cuffs, arrow heads, upper panel) and lesions reminiscent of diffuse alveolar damage (arrow heads, lower panel), characterized by fluid accumulation and inflammatory cell infiltration within the air space. Inoculation with PA103 (ΔPcrV) caused collapsed air spaces, resembling atelectasis with recruitment of inflammatory cells. These histological patterns suggest that the two PA103 mutants are capable to cause lung damage/injury, although the lesions appear distinct from one another. **B**. Injured areas of the lung were prepared for histological myeloperoxidase (MPO) staining analysis to reveal the extent of neutrophil infiltration. Inoculation with PA103 (ΔU/ΔT) caused significant neutrophil recruitment compared to either vehicle control or to inoculation with PA103 (ΔPcrV). The average numbers of MPO positive cells from at least 5 independent fields of view from three different animals per condition are presented. Error bars are the standard error of the mean. The asterisks indicate a statistically significant difference (*P* < 0.05) by one-way ANOVA and Newman-Keuls *post-hoc* test. **C**. Lungs from vehicle control (saline solution) and *P. aeruginosa*-inoculated animals were isolated *en* *bloc* at 48-hours post-inoculation for analysis of hydraulic permeability (K_f_). Inoculation with PA103 (ΔU/ΔT) caused significant permeability increases compared to either vehicle control or to inoculation with PA103 (ΔPcrV). The average K_f_ values from 5 different animals per group are shown. Error bars are the standard error of the mean. The asterisks indicate a statistically significant difference (*P* < 0.05) by one-way ANOVA and Newman-Keuls *post-hoc* test.

Macroscopic observation of lungs at 48-hours post-inoculation paralleled the mortality and morbidity observations described above, in that PA103 (ΔU/ΔT) caused more overt damage to the lung than PA103 (ΔPcrV) (Figure **S3**). We next compared fixed sections of the injured portions of lungs from rats inoculated with either PA103 (ΔU/ΔT) or PA103 (ΔPcrV) by hematoxylin and eosin (H&E) staining in survivors at 48-hours post-inoculation (both mutant strains were recovered from lung tissue at equivalent levels, data not shown). Figure **2A** shows H&E-stained sections imaged by light microscopy. The left panel set shows normal lung sections from an animal that underwent intra-tracheal instillation with saline solution (vehicle control). The septal network is thin and the airspaces are pristine. The center panel set shows lung sections from an animal infected with PA103 (ΔPcrV) which resulted in septal thickening and edema. The right panel set shows that infection with PA103 (ΔU/ΔT) resulted in thickening of the septal network and lung parenchyma, and the airspaces are either collapsed and/or appear edematous (stained proteinaceous fluid, marked with arrowheads in the 20X panel). Interestingly, infection with PA103 (ΔU/ΔT) resulted in formation of perivascular fluid-filled cuffs (marked with arrowheads in the 10X panel) which were not observed in PA103 (ΔPcrV)-infected animals or vehicle controls.

An independent set of lung sections were stained for myeloperoxidase to facilitate enumeration of infiltrating neutrophils as an indicator of inflammation. Figure **2B** shows that infection with PA103 (ΔU/ΔT) induced significant neutrophil infiltration compared to PA103 (ΔPcrV). This result supports observations made using a murine model of infection [36]. Together, these data suggest that the presence of a functional T3SS needle tip complex in PA103 (ΔU/ΔT) contributes to *P. aeruginosa*-induced lung injury.

Lung histopathology indicated that the presence of a functional T3SS needle tip complex was potentially influencing the extent of injury. Therefore, vascular permeability was quantitatively assessed by excising, *en bloc*, the heart and lungs from control and infected animals and measuring the fluid filtration coefficient (K_f_). In this method, the heart and lungs are suspended in a force transducer where buffer is perfused at a constant pressure over time and the rate of weight gain is used as a sensitive index of vascular endothelial cell barrier integrity (i.e., the extent of perfusate fluid leakage into the lung tissue) [64]. Figure **2C** shows that the lungs from animals infected with PA103 (ΔU/ΔT) displayed a significant increase in K_f_ compared to saline-treated or PA103 (ΔPcrV)-infected animals. These data suggest that the presence of a functional T3SS needle tip complex in PA103 (ΔU/ΔT) contributes to *P. aeruginosa*-induced lung injury in the absence of secreted effector proteins.

During the Early Phase of Infection, the P. *aeruginosa* PcrV Protein Contributes to PMVEC Barrier Disruption via Perturbation of the Host Actin Cytoskeleton that Occurs in a Cell Death-Independent Manner

The *in vivo* infection data presented thus far revealed a T3SS needle tip complex-dependent, but effector protein-independent mechanism by which *P. aeruginosa* pneumonia elicits hallmark features of ARDS, namely pulmonary edema (Figure **2A**), neutrophil infiltration (Figure **2B**), and increased fluid filtration (Figure **2C**). Importantly, these pathologies are all indicative of pulmonary endothelial barrier disruption. Thus, we tested an *in vitro* cell culture infection model to further compare the pathogenic potential of the T3SS needle tip complex to cause pulmonary endothelial barrier disruption and have revealed a two-phase, temporal nature of the pathogen-host interaction.

**Figure 3:**
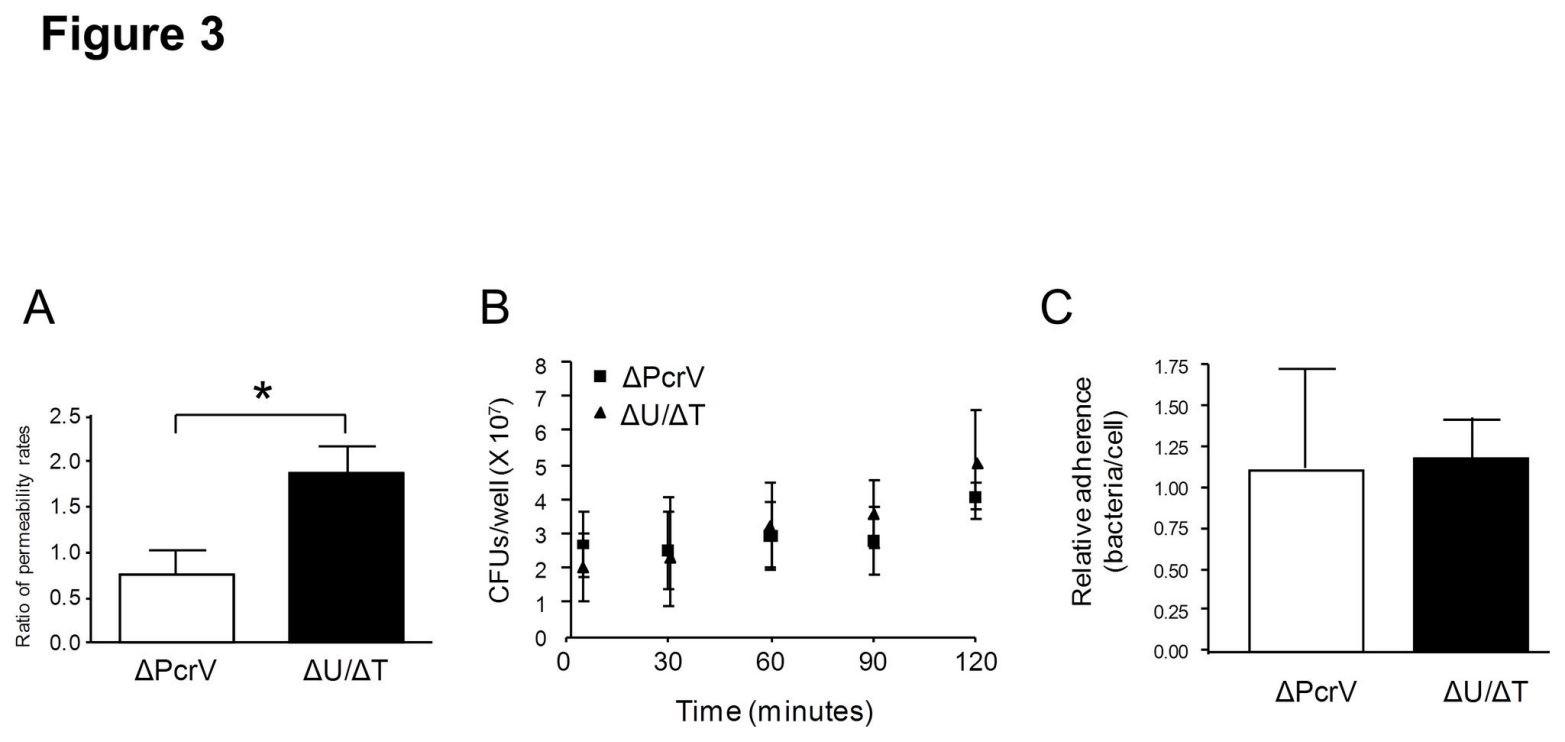
*P. aeruginosa* T3SS needle tip complex protein PcrV associates with increased PMVEC permeability during early phase infection. **A**. Isolated rat PMVECs were grown on transwells to confluence and permeability measured as a function of FITC-dextran flux across the monolayer. PMVECs were inoculated with medium alone, saline solution (vehicle control), or with either *P. aeruginosa* mutant at a 40:1 MOI. Flux assays were initiated at 4-hours post-inoculation by the addition of FITC-dextran and flux monitored for 1-hour. Average flux rate data at 5-hours post-inoculation from the SS vehicle control transwells were used to normalized the flux rate from PA103 (ΔU/ΔT) or PA103 (ΔPcrV) (n=3 for each condition). Inoculation with PA103 (ΔU/ΔT) caused significant increases in PMVEC permeability compared to inoculation with PA103 (ΔPcrV). Error bars are the standard error of the mean. The asterisk indicates a significant difference by unpaired *t*-test (*P* < 0.05). **B**. Mock cell culture infection experiments were performed where ~2.5 x 10^7^ CFU/mL of each *P. aeruginosa* mutant strain was inoculated into DMEM culture medium (no PMVECs) and bacterial growth followed over time by direct plating. Error bars are the standard error of the mean of technical replicates from at least 3 independent experiments. There were no observable differences in growth rates between the two *P. aeruginosa* mutant strains. **C**. The ability of each of the P. *aeruginosa* mutant strains to adhere to PMVECs was assessed. Isolated rat PMVECs were grown in culture dishes to confluence and inoculated with either *P. aeruginosa* mutant at a 40:1 MOI. At one hour post-inoculation, monolayers were washed copiously to remove planktonic bacteria, PMVECs permeabilized, and total associated bacteria determined by direct plating. Error bars are the standard error of the mean of technical replicates from at least 3 independent experiments. There were no observable differences in adherence to PMVECs between the two *P. aeruginosa* mutant strains.

We have previously described the isolation, propagation, and barrier-forming properties of PMVECs derived from rat lung capillaries [65,66] and herein used these cells to test the effects of *P. aeruginosa* infection. During the early phase of infection (5-hours post-inoculation), PA103 (ΔU/ΔT) induced significant PMVEC barrier disruption measured as an increased rate of fluorescein isothiocyanate-labeled dextran flux (FITC-dextran, 40,000 MW) across a confluent PMVEC monolayer adhered onto a transwell (Figure **3A**). Conversely, PMVECs inoculated with PA103 (ΔPcrV) demonstrated a rate of FITC-dextran flux comparable to uninoculated PMVEC controls (Figure **3A**).

Visual inspection of the monolayers by time-lapse phase contrast light microscopy was used as a second indicator of cell-to-cell association in confluent PMVEC monolayers during a time-course infection. The same region of the culture well was monitored with time and revealed that PMVECs inoculated with PA103 (ΔU/ΔT) begin to alter morphology during the early phase of infection (5-hours post-inoculation) compared to PMVECs inoculated with PA103 (ΔPcrV) and uninoculated PMVEC controls (Figure **S4**).

We have determined that the differences in PMVEC barrier disruption between the two *P. aeruginosa* strains were not due to differences in bacterial growth over the time course as both PA103 (ΔU/ΔT) and PA103 (ΔPcrV) displayed comparable growth rates (Figure **3B**). In addition, both PA103 (ΔU/ΔT) and PA103 (ΔPcrV) were equally able to adhere to the PMVEC monolayers indicating that the T3SS needle tip complex is not likely a major determining factor of pathogen-host attachment (Figure **3C**). Together, these data suggest that the T3SS needle tip complex contributes to *P. aeruginosa*-induced PMVEC barrier disruption during the early phase of infection.

**Figure 4:**
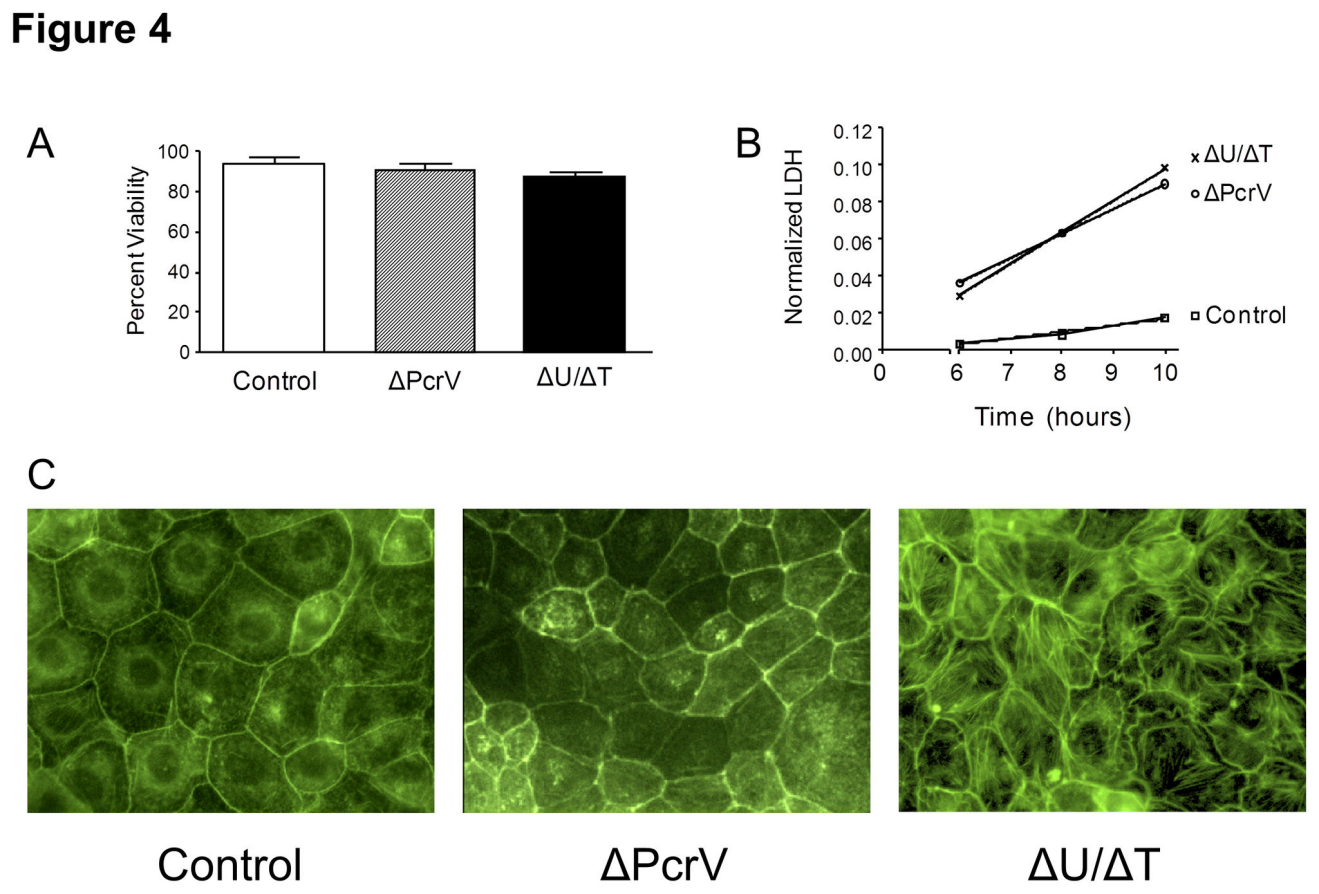
*P. aeruginosa* T3SS needle tip complex protein PcrV elicits increased PMVEC permeability during early phase infection via cytoskeletal perturbation in a host cell death-independent manner. **A**. Isolated rat PMVECs were grown in culture dishes to confluence and viability assessed by Trypan Blue exclusion assay. PMVECs were inoculated with saline solution (vehicle control), or with either *P. aeruginosa* mutant at a 40:1 MOI. PMVEC viability was assessed at 5-hours post-inoculation, and data are expressed as percent total viable cells. Neither PA103 mutant caused significant cell death compared to vehicle control-treated PMVECs. The average viable PMVEC numbers from at least 3 independent experiments per condition are presented. Error bars are the standard error of the mean. **B**. Isolated rat PMVECs were grown in culture dishes to confluence and damage assessed by LDH extracellular release assay. PMVECs were inoculated with saline solution (vehicle control), or with either *P. aeruginosa* mutant at a 40:1 MOI. PMVEC damage was assessed over a time course post-inoculation. Culture medium was sampled to measure extracellular LDH and data normalized to the total monolayer (intracellular) LDH. While measurable, compared to vehicle control-treated PMVECs, both PA103 mutants caused only negligible rates of LDH release. The average amounts of extracellular LDH from at least 3 independent experiments are presented. **C**. Isolated rat PMVECs were grown on cover slips to confluence and actin cytoskeleton was visualized by FITC-phalloidin staining. Z-stacks from whole cellular volume images were acquired via confocal fluorescence microscopy. PMVECs were inoculated with saline solution (vehicle control), or with either *P. aeruginosa* mutant at a 40:1 MOI. Inoculation with PA103 (ΔU/ΔT) mutant caused evident cytoskeletal rearrangement (stress fibers) compared to vehicle control and PA103 (ΔPcrV) inoculation.

Interestingly, the observed early phase of PMVEC barrier disruption induced by PA103 (ΔU/ΔT) was not attributable to infection-induced host cell death as measured by Trypan Blue exclusion assay (Figure **4A**), nor were there any measurable TUNEL-positive staining cells (data not shown). Inoculation of PMVECs with PA103 (ΔPcrV) also did not significantly affect host cell viability (Figure **4A** and data not shown). In addition, while both PA103 mutants caused measurable rates of LDH release, the amounts were negligible. (Figure **4B**). These data suggest that while PA103 (ΔU/ΔT) infection induces cell damage, the primary mechanism of early phase PMVEC barrier disruption occurs in a host cell death-independent manner.

Actin cytoskeletal rearrangements and perturbations have been implicated in other model systems as a critical, cell death-independent mechanism of PMVEC barrier disruption [67,68]. Thus, we examined actin in fixed cells by phalloidin staining and fluorescence confocal microscopy. Figure **4C** shows that inoculation of PMVECs with PA103 (ΔU/ΔT) resulted in actin stress fiber formation compared to PA103 (ΔPcrV) and uninoculated control cells. These data suggest that the T3SS needle tip complex induces early phase PMVEC barrier disruption via perturbation of the host cell actin cytoskeletal network.

### During the Late Phase of Infection, the P. *aeruginosa* PcrV Protein Contributes to PMVEC Barrier Disruption via Induction of Host Cell Damage and Death

**Figure 5:**
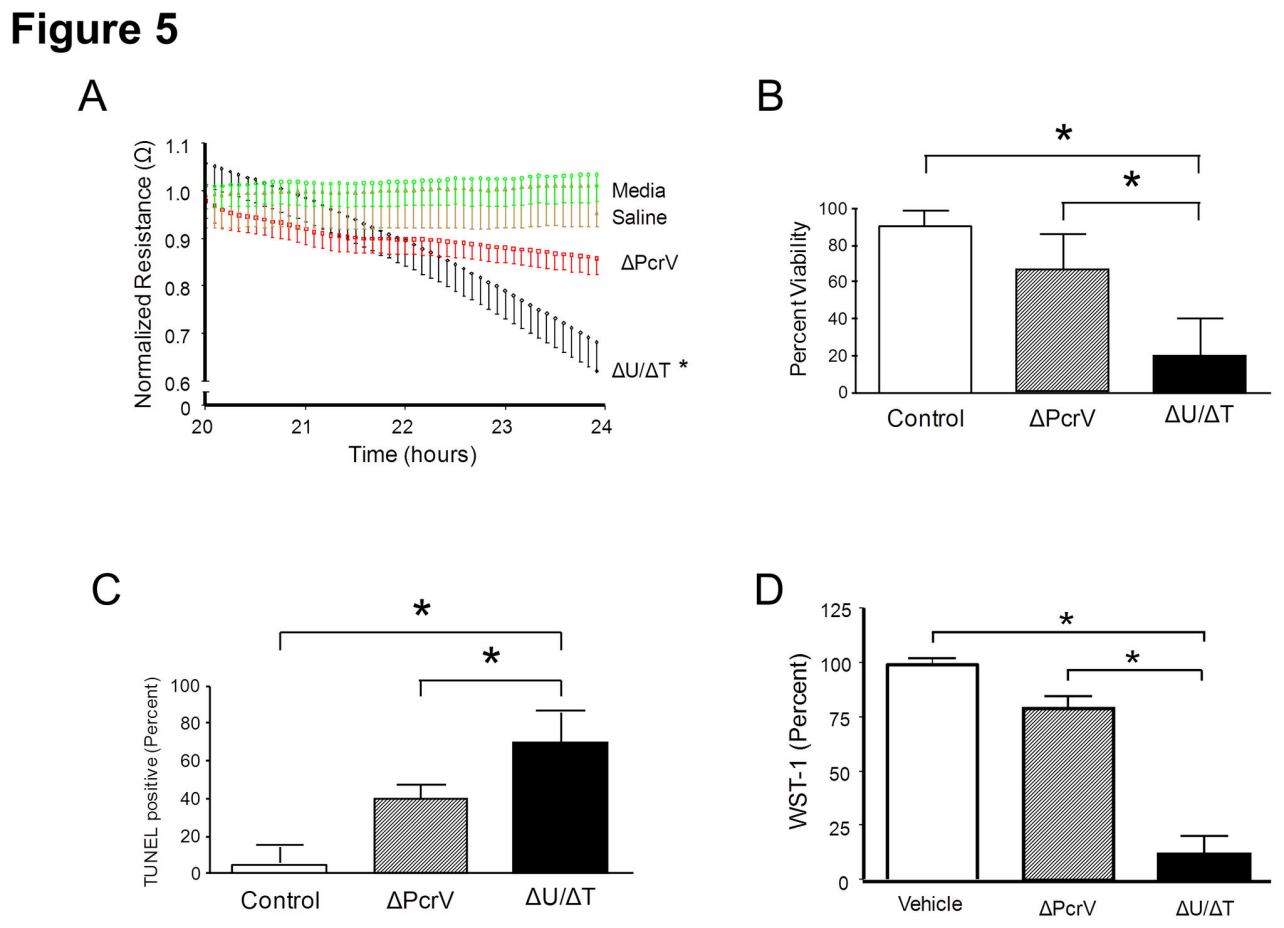
*P. aeruginosa* T3SS needle tip complex protein PcrV increases PMVEC permeability associated with cell damage during late phase infection. **A**. Isolated rat PMVECs were grown on gold electrodes to confluence and resistance across the monolayer determined using an ECIS system. PMVECs were inoculated with medium alone, saline solution (vehicle control), or with either *P. aeruginosa* mutant at a 40:1 MOI. Inoculation with PA103 (ΔU/ΔT) caused significant increases in PMVEC permeability (i.e., decreased electrical resistance) compared to no treatment (medium alone), vehicle control inoculation, or inoculation with PA103 (ΔPcrV). The average numbers of resistance recordings from at least 6 independent experiments per condition are presented. Error bars are the standard error of the mean. The asterisk indicates that PA103 (ΔU/ΔT) was significantly different compared to all other conditions (*P* < 0.05) by one-way ANOVA and Newman-Keuls *post-hoc* test. **B**. Isolated rat PMVECs were grown in culture dishes to confluence and viability assessed by Trypan Blue exclusion assay. PMVECs were inoculated with saline solution (vehicle control), or with either *P. aeruginosa* mutant at a 40:1 MOI. PMVEC viability was assessed at 24-hours post-inoculation, and data are expressed as percent total viable cells. Inoculation with PA103 (ΔU/ΔT) significantly increased PMVEC death compared to vehicle control inoculation and PA103 (ΔPcrV). The average viable PMVEC numbers from at least 10 independent experiments per condition are presented. Error bars are the standard error of the mean. The asterisks indicate that PA103 (ΔU/ΔT) was significantly different compared to all other conditions (*P* < 0.05) by one-way ANOVA and Newman-Keuls *post-hoc* test. **C**. Isolated rat PMVECs were grown on glass cover slips to confluence and cell death assessed by TUNEL staining. PMVECs were inoculated with saline solution (vehicle control), or with either *P. aeruginosa* mutant at a 40:1 MOI. PMVEC death was assessed at 24-hours post-inoculation, and numbers of dead cells are expressed as a percent of the total number of cells. Inoculation with PA103 (ΔU/ΔT) significantly increased PMVEC death compared to vehicle control inoculation and PA103 (ΔPcrV). Error bars are the standard error of the mean from 3 independent experiments. The asterisks indicate that PA103 (ΔU/ΔT) was significantly different compared to all other conditions (*P* < 0.05) by one-way ANOVA and Newman-Keuls *post-hoc* test. **D**. Isolated rat PMVECs were grown in culture dishes to confluence and cellular fitness assessed by WST-1 metabolism assay. PMVECs were inoculated with saline solution (vehicle control), or with either *P. aeruginosa* mutant at a 40:1 MOI. PMVECs were assessed at 24-hours post-exposure for each condition, and data are expressed as percent of the vehicle control. Infection with PA103 (ΔU/ΔT) significantly decreased PMVEC fitness compared to vehicle control inoculation and PA103 (ΔPcrV). The average amount of WST-1 metabolism from at least 5 independent experiments per condition is presented. Error bars are the standard error of the mean. The asterisks indicate that PA103 (ΔU/ΔT) was significantly different compared to all other conditions (*P* < 0.05) by one-way ANOVA and Newman-Keuls *post-hoc* test.

Extrapolation of the data in Figure **4B** suggests that inoculation with PA103 (ΔU/ΔT) will result in substantial host cell damage during the late phase of infection (24-hours post-inoculation), compared to PA103 (ΔPcrV). This observation is supported by Figure **5A** which shows the increased kinetics of PA103 (ΔU/ΔT)-induced PMVEC barrier disruption during the late phase of infection compared to PA103 (ΔPcrV), measured as a function of the transelectrical resistance (TER) of the monolayer.

Time-lapse phase contrast microscopic analysis of PMVEC monolayers revealed that during the late phase of infection PA103 (ΔU/ΔT) caused more PMVEC damage and loss of cell-to-cell adhesion compared to inoculation with PA103 (ΔPcrV) and uninoculated PMVEC controls (Figure **S4**). These data suggest that the presence of a functional T3SS needle tip complex in PA103 (ΔU/ΔT) contributes to late phase PMVEC barrier disruption and loss of cell-to-cell adhesion. These associated changes occurred over a protracted time course compared to what has been previously reported for strains that deploy the ExoU PLA_2_ secreted effector (we have confirmed this using the wild type PA103 strain, data not shown).

To better understand the mechanisms underlying the observed T3SS needle-tip complex-dependent barrier disruptive effects during the late phase of infection we examined PMVEC viability and metabolic function. Figure **5B** shows that PA103 (ΔU/ΔT) caused significantly more PMVEC death compared to inoculation with PA103 (ΔPcrV) and uninoculated PMVEC controls as assessed by Trypan Blue exclusion assay. Furthermore, analysis of cell death by TUNEL staining revealed a population of TUNEL-positive cells (Figures 5**C** and **S5**).

Finally, as an index of cellular fitness, PMVEC metabolic function was determined using WST-1, which measures cellular red-ox state. Figure **5D** shows that during the late phase of inoculation with a P. *aeruginosa* strain encoding a functional T3SS needle tip complex [PA103 (ΔU/ΔT)], the metabolic fitness of PMVECs is severely impaired compared to inoculation with PA103 (ΔPcrV) and uninoculated PMVEC controls. Together, these data suggest that the presence of a functional T3SS needle tip complex in PA103 (ΔU/ΔT) contributes to *P. aeruginosa*-induced barrier disruption via effects on PMVEC metabolic function and viability.

## Discussion

The studies presented herein describe a previously unappreciated role for the P. *aeruginosa* T3SS needle tip complex in the elicitation of pneumonia-induced lung injury and ARDS that occur independently of the four known secreted effector proteins. While the P. *aeruginosa* T3SS needle tip complex has been implicated as a virulence factor in animal infection models and in stimulating recruitment and cell death in immune cells to drive damaging inflammatory responses [4,21,44,45,51–53], ours is the first study to reveal the deleterious effects associated with lung endothelial barrier and systemic organ dysfunction.

Using a rat lung airway infection model, we have shown that a P. *aeruginosa* strain possessing a functional T3SS needle tip complex but devoid of the four known effector proteins is able to: 1) elicit neutrophil infiltration; 2) cause lung damage, alveolar edema/collapse, and increased permeability; and 3) cause damage sufficient to elicit dysfunction of at least two other organ systems. Deletion of the gene encoding the T3SS needle tip complex protein PcrV severely attenuated the three above-mentioned deleterious effects during infection of the lung.

Using a lung endothelial cell culture infection model, we have elucidated a two-phase, temporal nature of *P. aeruginosa* infection of PMVECs. Specifically, a P. *aeruginosa* strain possessing a functional T3SS but devoid of known effector proteins caused early phase barrier disruption (5-hours post-inoculation) by inducing actin cytoskeletal rearrangement, but without accompanying host cell death. A functional T3SS needle tip complex PcrV protein was essential to elicit early phase barrier disruption. Perturbations of the PMVEC actin cytoskeleton, including the formation of stress fibers as observed here, have been previously reported as an important determinant of PMVEC barrier disruption [67,68]. Future studies will be aimed at uncovering the host cell signaling pathways specifically disrupted or activated by a functional *P. aeruginosa* T3SS needle tip complex.

Hallmarks of late phase barrier disruption (24-hours post-inoculation) included damage and destruction of confluent PMVEC monolayers, loss of cell-to-cell adhesion, and were attributable to deleterious effects of infection on cellular fitness and viability. Deletion of the gene encoding the T3SS needle tip complex protein PcrV attenuated late phase barrier disruptive effects. The fact that there were high numbers of both, Trypan Blue- and TUNEL-positive cells suggested that PcrV-dependent cell death is a major contributor to PMVEC demise during late stages of infection. Thus, during the late stages of infection, cell-death dependent mechanisms elicited by the T3SS needle tip complex might be similar between PMVECs and immune cells where apoptosis was previously shown to be a major contributor to pathobiology in a murine infection model [36].

Our studies raise the intriguing questions of whether engagement of infected eukaryotic cells by the T3SS needle tip complex elicits damage directly or whether the translocation of unknown effector proteins is responsible for the observed deleterious effects. The damaging effects of the T3SS needle tip complex are inferred from red blood cell hemolysis assays describing its pore-forming capacity [55,69]. Interestingly, the extrapolated amount of LDH release from PMVECs during the late phase of infection suggested that the T3SS needle tip complex might be inducing cell damage and lysis. However, the reported T3SS needle tip complex-dependent hemolysis of red blood cells occurs over a much shorter time course than that required to damage PMVECs in our experiments and, thus, we can not entirely preclude a contribution of unknown effectors/hemolysins.

Another possible mechanism of T3SS needle tip complex-induced cellular damage/death during the late phase of infection is engagement of inflammasomes and activation of caspase-1. Families of germ-line encoded Pattern Recognition Receptor (PRR) proteins sense and respond to extracellular and intracellular signals that accompany infections [70–72]. In particular, the Nucleotide Oligomerization Domain-Like Receptors (NLRs) have emerged as important PRRs that mediate caspase-1 activation. Caspase-1 and NLRs assemble with scaffolding proteins to form inflammasomes – complexes that respond to environmental signals to stimulate caspase-1 activation. The T3SS itself has been shown to engage inflammasomes [73] in addition to bacterial proteins such as flagellin [70,73,74] (note that the PA103 strain used in our studies does not express flagellin). One consequence of caspase-1 activation is initiation of pyroptosis, a rapid form of programmed cell death described in immune cells [72]. However, there are no studies describing inflammasome function or caspase-1-mediated pyroptosis in PMVECs. We are currently examining this possibility in PMVECs and our preliminary, unpublished studies suggest that PMVEC inflammasome activation does not lead to induction of pyroptosis.

While mechanisms underlying the deleterious effects of the T3SS needle tip complex on lung and PMVEC function are unclear, the current evidence highlights an evolutionary importance of this molecular machinery in pathogen virulence. An extensive study of *P. aeruginosa* clinical isolates revealed that the presence of a T3SS and either of the ExoU or ExoS effector proteins correlated with severity of outcome [4]. Interestingly, clinical isolates encoding only PcrV correlated with more severe outcomes compared to patients infected with strains lacking a functional T3SS. Together these data suggest a possible pathway of evolutionary acquisition of virulence factors that were subverted from their original function in bacteria cellular physiology to facilitate colonization of new niches (such as humans). Just as the restriction endonuclease systems likely evolved to protect the bacterium from invading foreign nucleic acids, perhaps the ancestral T3SS endowed a selective advantage by jettisoning potentially toxic protein products encoded by invading foreign genetic elements. This idea is suggested by the fact that many examples of T3SSs and their effectors reside in pathogenicity islands which tend to possess a different AT:GC ratio than the core bacterial genome, suggesting their acquisition via horizontal gene transfer. These intriguing evolutionary questions and the clinical relevance to combating *P. aeruginosa* pneumonia-induced ARDS underscore the need for future studies directed at furthering our understanding of the direct role of the T3SS needle tip complex in pathogen-host interactions and cellular viability/damage. Furthermore, it will be necessary to determine whether *P. aeruginosa* isolates expressing the ExoS effector (i.e., non-PA103-like strains) also employ the T3SS needle tip complex as an effector-independent virulence factor.

## Materials and Methods

### Ethics Statement

 This study was carried out in accordance with the recommendations in the Guide for the Care and Use of Laboratory Animals of the National Institutes of Health. The experimental protocol was approved by the University of South Alabama IACUC (protocol number 278188). All surgeries were performed after confirming that animals had achieved anesthetic plane and all efforts were made to minimize suffering. For the survival studies described herein, animals were humanely euthanized when they exhibited signs of high respiratory distress or by direct recommendation of the veterinarian. For all other studies, control and infected animals were humanely euthanized within 72-hours post inoculation. Animals were euthanized with a barbiturate overdose followed by exsanguination, a procedure conforming to recommendations from the veterinarian panel of euthanasia and the IACUC.

### Bacterial Strains and Growth

 The P. *aeruginosa* strains used in this study were kindly provided by Dr. Dara Frank (Medical College of Wisconsin). The wild type PA103 is a clinical isolate that expresses a functional T3SS and the effector proteins ExoU and ExoT (PA103 does not encode the effector proteins ExoS and ExoY). PA103 (ΔU/ΔT) is an isogenic PA103 mutant deleted for *exoU* and possessing a tetracycline resistance cassette inserted into *exoT* (PA103 *ΔexoU:exoT*::Tc) but expresses a fully functional T3SS [75]. PA103 (ΔPcrV) is an isogenic PA103 mutant that possesses functional *exoU* and *exoT* genes but is deleted for the *pcrV* gene (PA103 *ΔpcrV*) and, thus cannot form a functional T3SS needle tip complex. PA103 (ΔPcrV) is unable to inject ExoU and ExoT into eukaryotic host cells and instead, the effector proteins are secreted indiscriminately into the culture medium [63].

 Bacteria from frozen stock solutions (Difco Nutrient Broth medium with 15% glycerol, stored at -80 °C) were routinely cultured on the minimal E salts medium of Vogel and Bonner overnight at 37 °C. Immediately prior to use in infection experiments, bacteria were washed in 10 mL of normal saline solution (SS), collected by centrifugation (4000 x*g*, 10 minutes, at room temperature), and suspended in SS. The correlation between OD 600_nm_ and relative CFU/mL for all three bacterial strains was determined by serial dilution and direct plate counts on Luria Bertani (LB) agar (grown overnight at 37 °C), and spectrophotometry used for CFU/mL determinations in animal and cell culture infection experiments thereafter.

### Rat Airway Infection Experiments

 Adult, male CD rats were purchased from Charles River Laboratories. Animals weighing between 275 - 300 grams were anesthetized with ketamine/xylazine (75/5 mg/kg intra-peritoneally, respectively). After confirming anesthetic plane, the ventral neck area of the animal was prepared with 70 % ethanol, an incision of 2 cm length was made, the sternomastoid muscles were retracted, and the trachea exposed. The animal was positioned at a 75^O^ Fowler’s angle and a 27-gauge needle inserted in between tracheal rings immediately below the thyroid gland to inject 150 µL SS delivered caudally. After 3-minutes post inoculation, the skin was sutured and the animal transferred to a clean cage for recovery. These were considered control, sham surgery animals. Infected animals underwent the same procedure except that the 150 µL SS contained any one of the three aforementioned *P. aeruginosa* strains (prepared as described above) at the concentrations described in the results section. This protocol was used to generate the one-week survival dose response and Kaplan-Meier curves.

 At 48-hours post-infection, body temperature was measured rectally in anesthetized animals. A microtipped pressure-volume catheter (1.4 Fr, Millar instruments) was advanced through the carotid artery and used to measure ventricular function (dP/dt). Peripheral blood was subsequently collected and analyzed for total and differential white cell counts, aspartate aminotransferase (AST), and creatinine. CFUs were determined from peripheral blood and lung tissue by direct plate counts on Luria Bertani (LB) agar (grown overnight at 37 °C).

### Lung Histopathology

 Terminal experiments were performed in control or infected animals anesthetized as described above. Animals were mechanically ventilated (Harvard Inspira 55-7058) with 6 mL of room air per kg body weight at 55 breaths per minute and 2 cmH_2_O positive end expiratory pressure. Following a thoracotomy, the trachea was tied-off at the peak of inspiration, the lungs removed, and then immersed in 10 % neutral buffered formalin solution. Lung specimens were embedded in paraffin and processed for hematoxylin and eosin (H&E) staining. Sections at 5 µm thickness were visualized on a Nikon coupled to a CCD camera and images acquired at 10x and 20x magnification.

An independent set of lung sections from the same animals were stained for myeloperoxidase (MPO) to facilitate enumeration of infiltrating neutrophils following the protocol previously described in [76]. The average numbers of MPO positive cells from at least 5 independent fields of view from three different animals per condition are presented.

### Lung Hydraulic Permeability

 Terminal experiments were performed in control or infected animals anesthetized and ventilated as described above. Following a thoracotomy, the pulmonary artery and the left atrium were cannulated and constant perfusion initiated with Earle’s Buffered Salt Solution containing 4% bovine serum albumin (EBSS 4% BSA). The solution pH was adjusted to 7.4 using NaHCO_3_^-^. The lung and heart were removed *en bloc*, and suspended in a force transducer. Pulmonary venous and arterial pressures, and lung weight were digitally recorded using a Biopac System (AD Instruments). Flow rates were adjusted based on animal weight and venous pressure set at 5 cmH_2_O [64]. After 10 minutes of perfusion, pulmonary capillary pressure was measured (baseline) via double occlusion, venous pressure was then raised to 15 cmH_2_O for 15 minutes and capillary pressure measured to calculate its change compared to baseline. Lung hydraulic permeability is calculated as the normalized rate of lung weight gain over the change in capillary pressure during the last 2 minutes of elevated venous pressure and expressed as K_f_.

### Propagation and Infection of PMVECs *in vitro* for Barrier Assays

 PMVECs from CD rats were isolated and characterized as previously described [65], and made available for our studies through the University of South Alabama Center for Lung Biology Cell Culture Core. Low passage number cells (<15) were routinely cultured in DMEM high glucose medium (Invitrogen) containing 10% fetal bovine serum (DMEM/FBS) and grown at 37 °C, (5% CO_2_, atm O_2_) to confluence prior to infection.

 The early phase effects (5-hours post-inoculation) of the T3SS needle tip complex on PMVEC barrier function were examined using a fluorescein isothiocyanate (FITC)-dextran flux assay. PMVECs in DMEM culture medium containing 1% FBS were seeded onto polycarbonate transwell filters (0.4 µm pore size, 6.5 mm insert) at 1.3 x 10^5^ cells per well and incubated overnight to give a final cell density of ~2.6 x 10^5^ cells per well (confluence) at the time of inoculation. *P. aeruginosa* PA103 (ΔU/ΔT) or PA103 (ΔPcrV) were then added to individual wells to achieve a multiplicity of infection (MOI) of 40 bacteria: 1 PMVEC (SS was added to individual wells as the vehicle control). At 4-hours post inoculation, FITC-dextran (40,000 MW from Sigma, 0.1 mg/mL in DMEM 1% FBS culture medium) was added to each transwell chamber. The transwell chambers were then moved to new bottom wells containing fresh DMEM 1% FBS culture medium and samples removed at 15-, 30-, 45-, and 60-minutes, and stored protected from light until the end of the assay. The amount of FITC-dextran flux across the monolayer was determined by measuring fluorescent signal from the medium in the bottom well on a NanoDrop Fluorospectrometer ND-330 (ThermoScientific) using an excitation wavelength of 488 nm +/- 10 nm and an emission wavelength of 515 nm. Increases in the rate of FITC-dextran flux across the monolayer are indicative of PMVEC barrier disruption. Average flux rate data from the SS vehicle control transwells were used to normalized the flux rate from duplicate inoculations with PA103 (ΔU/ΔT) or PA103 (ΔPcrV) (n=3 for each condition).

The late phase effects (24-hours post-inoculation) of the T3SS needle tip complex on PMVEC barrier function were examined using the transelectrical resistance (TER) assay. PMVECs were seeded onto gold electrodes at 4.5 x 10^4^ cells per well, grown for 2-days to a final concentration of 4.0 x 10^5^ cells per well (confluence), and the baseline resistance across the monolayer determined using the Electric Cell-substrate Impedance Sensing system (Applied Biophysics) as previously described [77]. *P. aeruginosa* PA103 (ΔU/ΔT) or PA103 (ΔPcrV) at an MOI of 40:1 were then added to individual wells and monolayer resistance monitored in real time over a 24-hour time course (SS was added to individual wells as the vehicle control). The resistance of the confluent monolayer prior to challenge was used to normalize all subsequent resistance values acquired post-challenge. A decrease in resistance with time is indicative of barrier disruptive effects of infection.

 To examine PMVEC barrier function by time-lapse phase contrast microscopy, cells were seeded as described above. Medium was removed from the well and replaced with DMEM without FBS followed by addition of bacteria to individual wells at a MOI of 40:1 (SS was added to individual wells as a vehicle control). A single region of the culture well was marked and images from the same area were serially captured by phase contrast microscopy over a 24-hour time course. Still images are shown at early phase infection (5-hours post-inoculation) and at late phase infection (24-hours post-inoculation). Increased PMVEC rounding, appearance of intercellular gap formation (loss of cell-to-cell adhesion), and appearance of fully exposed areas due to cell sloughing are indicators of the barrier disruptive effects of infection.

### *P. aeruginosa* Growth Rates and Adherence to PMVECs *in vitro*

 Growth rate of *P. aeruginosa* strains under the static cell culture infection conditions was determined by removing aliquots over time and counting bacteria by serial dilution in SS and direct plate counts on LB agar (grown overnight at 37 °C).

 Adherence of *P. aeruginosa* strains to PMVECs during cell culture infection experiments was determined at 1-hour post-infection. At that time, the culture medium was removed and wells washed 2-times with 5 mL of DMEM. A third wash with 1 mL of DMEM was performed and 0.1 mL of this wash solution plated onto LB agar to assess the efficiency of removing the unattached bacteria. Finally, monolayers were lysed with 1 mL of 0.1% Triton X-100 to release all associated bacteria. Bacterial numbers were determined in triplicate by direct plate counts of 0.1 mL of lysate onto LB agar (grown overnight at 37 °C). We also verified that the addition of 0.1% Triton X-100 had no adverse effects on *P. aeruginosa* plating efficiency.

### Infection of PMVECs *in vitro* for Viability Assays

To examine changes in cell viability at early (5-hours post-inoculation) phase of infection, PMVECs were grown to confluence and inoculated as described above. At the times indicated, medium was removed, wells washed with phosphate buffered saline (PBS, Invitrogen), incubated for 5-minutes with trypsin-EDTA (Invitrogen) to free cells from the well, the trypsin-EDTA removed by washing in DMEM/FBS and centrifugation (500 x*g*, 4-minutes, at room temperature), cells stained with Trypan Blue, and live/dead cells enumerated using a Countess Automated Cell Counter as per the manufacturer’s directions (Invitrogen). Only the dead cells take up the dye. The numbers of live and dead cells in the SS vehicle control wells were used to normalize data from the various infection conditions.

As a second measure of cellular death, TUNEL staining was performed on PMVECs grown and inoculated with saline solution (vehicle control), PA103 (ΔPcrV), or PA103 (ΔU/ΔT) as above except that cells were adhered to glass cover slips placed sterilely onto the bottom of the culture well. At 5- or 24-hours post-inoculation, PMVECs were permeabilized in phosphate buffered saline solution (PBS) containing 0.15% Triton X-100. TUNEL staining (indicative of late apoptotic cells) was performed using FITC-conjugated TUNEL labeling reagent following the manufacturer’s instructions (Roche). After TUNEL staining, slides were incubated with antibody against PECAM-1 (CD31), washed in PBS, and then incubated with an Alexa Fluor560-conjugated secondary antibody. Images were acquired by confocal fluorescent microscopy using a Nikon A1 equipped for hyperspectral imaging [78]. Post-acquisition image analysis for quantification of TUNEL-positive cells was performed using Cell Profiler cell image analysis software (Broad Institute) where 9 consecutive fields of view per slide were stitched together and the total number of TUNEL-positive cells normalized to the total number of PECAM-1-positive cells. Data were collected in this manner from 3 independent experiments.

### Infection of PMVECs *in vitro* for Visualization of the Actin Cytoskeleton

 To examine changes in the host cell actin cytoskeleton, phalloidin staining was performed on PMVECs grown and inoculated with saline solution (vehicle control), PA103 (ΔPcrV), or PA103 (ΔU/ΔT) as above except that cells were adhered to glass coverslips. At 5-hours post-inoculation, PMVECs were permeabilized in PBS containing 0.05% saponin, washed in PBS, incubated in FITC-labeled phalloidin (Life Technologies), washed in PBS, and fixed using chilled methanol (-20 °C). Slides were mounted using Antifade mounting medium (Dako) and images were acquired by confocal fluorescent microscopy using a Nikon A1. Z-stack images encompassing the entire cell volume were acquired and compressed. Data were collected in this manner from 3 independent experiments.

### Infection of PMVECs *in vitro* for Damage and Metabolism Assays

To examine changes in cellular damage, PMVECs were grown to confluence and inoculated with saline solution (vehicle control), PA103 (ΔPcrV), or PA103 (ΔU/ΔT). At the times indicated, culture medium was serially removed and the amount of host cell lactate dehydrogenase (LDH) released was assayed using a cytotoxicity detection kit as per the manufacturer’s directions (Roche). The amount of LDH detected in the culture medium was normalized to the total cellular LDH in lysed, uninfected control monolayers. Data were collected in this manner from 3 independent experiments.

 To examine changes in the cellular red-ox state in response to infection the PMVEC culture infection experiments described above were scaled down to a 96-well format. At 24-hours post-infection, the WST-1 reagent was added to the wells for 1-hour and visualized by spectrophotometry as per the manufacturer’s direction (Roche). Growing cells metabolize the WST-1 dye resulting in proportional changes in medium color. The extent of WST-1 metabolism in the infected wells was normalized to the SS vehicle control wells. Data were collected in this manner from 5 independent experiments.

### Statistical Analyses

 Data are reported as mean ± standard error. GraphPad Prism v4.3 was used for all analyses. Comparison among groups was performed using parametric analyses. Unpaired *t*-test was used to compare two groups. One-way ANOVA was used to compare more than two groups with a Newman-Keuls *post-hoc* test applied as necessary. Differences with a P value < 0.05 were considered significant.

## Supporting Information

Figure S1 ***P. aeruginosa* infection causes liver injury in a rat model of lung infection.** Histological hematoxylin and eosin image of control liver and from animals inoculated with PA103, PA103 (ΔPcrV), and with PA103 (ΔU/ΔT). Arrows point to regions with significant peri-portal inflammation only in animals inoculated with the wild type PA103 and PA103 (ΔU/ΔT) indicating that the presence of a functional T3SS needle tip complex is sufficient to elicit injury. These data are consistent with the observed elevated biomarkers of liver injury (see Table **1**).
(TIF)

Figure S2 ***P. aeruginosa* infection causes kidney injury in a rat model of lung infection.** Histological hematoxylin and eosin image of control kidney and from animals inoculated with PA103, PA103 (ΔPcrV), and with PA103 (ΔU/ΔT). White arrows point to glomeruli as a point of reference. Black arrows point to tubular cast indicative of sepsis-induced injury that was observed only in animals inoculated with the wild type PA103.
(TIF)

Figure S3 ***P. aeruginosa* T3SS needle tip complex protein PcrV associates with lung injury.** Macroscopic images of lungs at 48-hours post-inoculation. The left panel shows digital images of lungs from an animal inoculated with saline solution (vehicle control), the middle panel from an animal inoculated with ~1 x 10^7^ CFUs of PA103 (ΔPcrV), and the right panel from an animal inoculated with ~1 x 10^7^ CFUs of PA103 (ΔU/ΔT). PA103 (ΔU/ΔT) caused more gross damage to the lung compared to PA103 (ΔPcrV).
(TIF)

Figure S4 ***P. aeruginosa* T3SS needle tip complex protein PcrV associates with PMVEC barrier disruption.** Isolated rat PMVECs were grown in culture dishes to confluence and monolayer integrity was assessed by time-lapsed light microscopy. PMVECs were inoculated with saline solution (Vehicle control), or with either *P. aeruginosa* mutant at a 40:1 MOI. Exposures of the same region of the culture dish were obtained pre-exposure, at 5-6-hours, and at 24-hours post-inoculation. PMVECs inoculated with PA103 (ΔU/ΔT) begin to alter morphology during the early phase of infection (5-hours post-inoculation) compared to PMVECs inoculated with PA103 (ΔPcrV) and uninoculated PMVEC controls. During the late phase of infection PA103 (ΔU/ΔT) caused more PMVEC damage and loss of cell-to-cell adhesion compared to inoculation with PA103 (ΔPcrV) and uninoculated PMVEC controls. The PA103 (ΔPcrV) mutant also caused barrier disruption, but in a protracted fashion. Representative images are shown. Experiments were repeated at least 10 times per condition.
(TIF)

Figure S5 ***P. aeruginosa* T3SS needle tip complex protein PcrV associates with PMVEC cell death during the late phase of infection.** Isolated rat PMVECs were grown on glass cover slips to confluence and cell death was assessed by TUNEL staining and visualized via confocal fluorescence microscopy and hyperspectral imaging. PMVECs were inoculated with saline solution (vehicle control), or with either *P. aeruginosa* mutant at a 40:1 MOI. Slides were prepared for staining at 24-hours post-inoculation. PMVECs were stained with antibody against PECAM-1 (CD31, green pseudocolor) and TUNEL positive cells shown in red pseudocolor. PMVECs inoculated with PA103 (ΔU/ΔT) display significant number of TUNEL-positive cells compared to PMVECs inoculated with PA103 (ΔPcrV) and uninoculated PMVEC controls.
(TIF)

## References

[1] Chastre J, Fagon JY (2002) Ventilator-associated pneumonia. Am J Respir Crit Care Med 165: 867-903. doi:10.1164/ajrccm.165.7.2105078. PubMed: 11934711. 11934711

[2] Kurahashi K, Kajikawa O, Sawa T, Ohara M, Gropper MA et al. (1999) Pathogenesis of septic shock in *Pseudomonas* *aeruginosa* pneumonia. J Clin Invest 104: 743-750. doi:10.1172/JCI7124. PubMed: 10491409. 10491409 PMC408437

[3] Lynch JP III (2001) Hospital-acquired pneumonia: risk factors, microbiology, and treatment. Chest 119: 373S-384S. doi:10.1378/chest.119.2_suppl.373S. PubMed: 11171773. 11171773

[4] Roy-Burman A, Savel RH, Racine S, Swanson BL, Revadigar NS et al. (2001) Type III protein secretion is associated with death in lower respiratory and systemic *Pseudomonas* *aeruginosa* infections. J Infect Dis 183: 1767-1774. doi:10.1086/320737. PubMed: 11372029. 11372029

[5] Sandiumenge A, Rello J (2012) Ventilator-associated pneumonia caused by ESKAPE organisms: cause, clinical features, and management. Curr Opin Pulm Med 18: 187-193. doi:10.1097/MCP.0b013e328351f974. PubMed: 22366995. 22366995

[6] Yu P, Martin CM (2000) Increased gut permeability and bacterial translocation in *Pseudomonas* pneumonia-induced sepsis. Crit Care Med 28: 2573-2577. PubMed: 10921597. 10921597 10.1097/00003246-200007000-00065

[7] Allmond LR, Ajayi T, Moriyama K, Wiener-Kronish JP, Sawa T (2004) V-antigen genotype and phenotype analyses of clinical isolates of *Pseudomonas* *aeruginosa* . J Clin Microbiol 42: 3857-3860. doi:10.1128/JCM.42.8.3857-3860.2004. PubMed: 15297549. 15297549 PMC497603

[8] Feltman H, Schulert G, Khan S, Jain M, Peterson L et al. (2001) Prevalence of type III secretion genes in clinical and environmental isolates of *Pseudomonas* *aeruginosa* . Microbiology 147: 2659-2669. PubMed: 11577145. 11577145 10.1099/00221287-147-10-2659

[9] Garau J, Gomez L (2003) *Pseudomonas* *aeruginosa* pneumonia. Curr Opin Infect Dis 16: 135-143. doi:10.1097/00001432-200304000-00010. PubMed: 12734446. 12734446

[10] Lee VT, Smith RS, Tümmler B, Lory S (2005) Activities of *Pseudomonas* *aeruginosa* effectors secreted by the Type III secretion system *in* *vitro* and during infection. Infect Immun 73: 1695-1705. doi:10.1128/IAI.73.3.1695-1705.2005. PubMed: 15731070. 15731070 PMC1064929

[11] Azghani AO, Idell S, Bains M, Hancock RE (2002) *Pseudomonas* *aeruginosa* outer membrane protein F is an adhesin in bacterial binding to lung epithelial cells in culture. Microb Pathog 33: 109-114. doi:10.1006/mpat.2002.0514. PubMed: 12220987. 12220987

[12] Hassett DJ, Korfhagen TR, Irvin RT, Schurr MJ, Sauer K et al. (2010) *Pseudomonas* *aeruginosa* biofilm infections in cystic fibrosis: insights into pathogenic processes and treatment strategies. Expert Opin Ther Targets 14: 117-130. doi:10.1517/14728220903454988. PubMed: 20055712. 20055712

[13] Rybtke MT, Jensen PO, Høiby N, Givskov M, Tolker-Nielsen T et al. (2011) The implication of *Pseudomonas* *aeruginosa* biofilms in infections. Inflamm Allergy Drug Targets 10: 141-157. doi:10.2174/187152811794776222. PubMed: 21314623. 21314623

[14] Heiniger RW, Winther-Larsen HC, Pickles RJ, Koomey M, Wolfgang MC (2010) Infection of human mucosal tissue by *Pseudomonas* *aeruginosa* requires sequential and mutually dependent virulence factors and a novel pilus-associated adhesin. Cell Microbiol 12: 1158-1173. doi:10.1111/j.1462-5822.2010.01461.x. PubMed: 20331639. 20331639 PMC2906647

[15] Hritonenko V, Mun JJ, Tam C, Simon NC, Barbieri JT et al. (2011) Adenylate cyclase activity of *Pseudomonas* *aeruginosa* ExoY can mediate bleb-niche formation in epithelial cells and contributes to virulence. Microb Pathog 51: 305-312. doi:10.1016/j.micpath.2011.08.001. PubMed: 21843628. 21843628 PMC3213052

[16] Zaas DW, Swan ZD, Brown BJ, Li G, Randell SH et al. (2009) Counteracting signaling activities in lipid rafts associated with the invasion of lung epithelial cells by *Pseudomonas* *aeruginosa* . J Biol Chem 284: 9955-9964. doi:10.1074/jbc.M808629200. PubMed: 19211560. 19211560 PMC2665119

[17] Ader F, Le Berre R, Faure K, Gosset P, Epaulard O et al. (2005) Alveolar response to *Pseudomonas* *aeruginosa*: role of the type III secretion system. Infect Immun 73: 4263-4271. doi:10.1128/IAI.73.7.4263-4271.2005. PubMed: 15972518. 15972518 PMC1168600

[18] Hauser AR, Cobb E, Bodi M, Mariscal D, Vallés J et al. (2002) Type III protein secretion is associated with poor clinical outcomes in patients with ventilator-associated pneumonia caused by *Pseudomonas* *aeruginosa* . Crit Care Med 30: 521-528. doi:10.1097/00003246-200203000-00005. PubMed: 11990909. 11990909

[19] Auvin S, Collet F, Gottrand F, Husson MO, Leroy X et al. (2005) Long-chain polyunsaturated fatty acids modulate lung inflammatory response induced by *Pseudomonas* *aeruginosa* in mice. Pediatr Res 58: 211-215. doi:10.1203/01.PDR.0000169979.27641.40. PubMed: 16085793. 16085793

[20] Boyer S, Faure K, Ader F, Husson MO, Kipnis E et al. (2005) Chronic pneumonia with *Pseudomonas* *aeruginosa* and impaired alveolar fluid clearance. Respir Res 6: 17. doi:10.1186/1465-9921-6-17. PubMed: 15707485. 15707485 PMC551591

[21] Diaz MH, Hauser AR (2010) *Pseudomonas* *aeruginosa* cytotoxin ExoU is injected into phagocytic cells during acute pneumonia. Infect Immun 78: 1447-1456. doi:10.1128/IAI.01134-09. PubMed: 20100855. 20100855 PMC2849415

[22] Faure K, Sawa T, Ajayi T, Fujimoto J, Moriyama K et al. (2004) TLR4 signaling is essential for survival in acute lung injury induced by virulent *Pseudomonas* *aeruginosa* secreting type III secretory toxins. Respir Res 5: 1. doi:10.1186/1465-9921-5-1. PubMed: 15040820. 15040820 PMC389879

[23] Brigham KL, Woolverton WC, Blake LH, Staub NC (1974) Increased sheep lung vascular permeability caused by *pseudomonas* bacteremia. J Clin Invest 54: 792-804. doi:10.1172/JCI107819. PubMed: 4430713. 4430713 PMC301619

[24] Bucior I, Mostov K, Engel JN (2010) Pseudomonas aeruginosa-mediated damage requires distinct receptors at the apical and basolateral surfaces of the polarized epithelium. Infect Immun 78: 939-953. doi:10.1128/IAI.01215-09. PubMed: 20008530. 20008530 PMC2825949

[25] Eutamene H, Theodorou V, Schmidlin F, Tondereau V, Garcia-Villar R et al. (2005) LPS-induced lung inflammation is linked to increased epithelial permeability: role of MLCK. Eur Respir J 25: 789-796. doi:10.1183/09031936.05.00064704. PubMed: 15863634. 15863634

[26] Ganter MT, Roux J, Su G, Lynch SV, Deutschman CS et al. (2009) Role of small GTPases and alphavbeta5 integrin in *Pseudomonas* *aeruginosa*-induced increase in lung endothelial permeability. Am J Respir Cell Mol Biol 40: 108-118. doi:10.1165/rcmb.2007-0454OC. PubMed: 18703797. 18703797 PMC2606944

[27] Lange M, Hamahata A, Enkhbaatar P, Esechie A, Connelly R et al. (2008) Assessment of vascular permeability in an ovine model of acute lung injury and pneumonia-induced *Pseudomonas* *aeruginosa* sepsis. Crit Care Med 36: 1284-1289. doi:10.1097/CCM.0b013e318169ef74. PubMed: 18379256. 18379256

[28] Aird WC (2007) Phenotypic heterogeneity of the endothelium: II. Representative vascular beds. Circ Res 100: 174-190. Available online at: doi:10.1161/01.RES.0000255690.03436.ae. PubMed: 17272819. 17272819 10.1161/01.RES.0000255690.03436.ae

[29] Aird WC (2007) Phenotypic heterogeneity of the endothelium: I. Structure, function, and mechanisms. Circ Res 100: 158-173. doi:10.1161/01.RES.0000255691.76142.4a. PubMed: 17272818. 17272818

[30] Alvarez DF, Huang L, King JA, ElZarrad MK, Yoder MC et al. (2008) Lung microvascular endothelium is enriched with progenitor cells that exhibit vasculogenic capacity. Am J Physiol Lung Cell Mol Physiol 294: L419-L430. PubMed: 18065657. 18065657 10.1152/ajplung.00314.2007

[31] Cines DB, Pollak ES, Buck CA, Loscalzo J, Zimmerman GA et al. (1998) Endothelial cells in physiology and in the pathophysiology of vascular disorders. Blood 91: 3527-3561. PubMed: 9572988. 9572988

[32] Dudek SM, Jacobson JR, Chiang ET, Birukov KG, Wang P et al. (2004) Pulmonary endothelial cell barrier enhancement by sphingosine 1-phosphate: roles for cortactin and myosin light chain kinase. J Biol Chem 279: 24692-24700. doi:10.1074/jbc.M313969200. PubMed: 15056655. 15056655

[33] Garcia AN, Vogel SM, Komarova YA, Malik AB (2011) Permeability of endothelial barrier: cell culture and *in* *vivo* models. Methods Mol Biol 763: 333-354. doi:10.1007/978-1-61779-191-8_23. PubMed: 21874463. 21874463

[34] Garcia JG, Verin AD, Schaphorst K, Siddiqui R, Patterson CE et al. (1999) Regulation of endothelial cell myosin light chain kinase by Rho, cortactin, and p60(src). Am J Physiol 276: L989-L998. PubMed: 10362724. 10362724 10.1152/ajplung.1999.276.6.L989

[35] Lowe K, Alvarez D, King J, Stevens T (2007) Phenotypic heterogeneity in lung capillary and extra-alveolar endothelial cells. Increased extra-alveolar endothelial permeability is sufficient to decrease compliance. J Surg Res 143: 70-77. doi:10.1016/j.jss.2007.03.047. PubMed: 17950075. 17950075 PMC6750899

[36] Galle M, Jin S, Bogaert P, Haegman M, Vandenabeele P et al. (2012) The *Pseudomonas* *aeruginosa* type III secretion system has an exotoxin S/T/Y independent pathogenic role during acute lung infection. PLOS ONE 7: e41547. doi:10.1371/journal.pone.0041547. PubMed: 22844497. 22844497 PMC3402384

[37] Ware LB, Matthay MA (2000) The acute respiratory distress syndrome. N Engl J Med 342: 1334-1349. doi:10.1056/NEJM200005043421806. PubMed: 10793167. 10793167

[38] Ware LB, Matthay MA (2005) Clinical practice. Acute pulmonary edema. N Engl J Med 353: 2788-2796. doi:10.1056/NEJMcp052699. PubMed: 16382065. 16382065

[39] El-Solh AA, Hattemer A, Hauser AR, Alhajhusain A, Vora H (2012) Clinical outcomes of type III *Pseudomonas* *aeruginosa* bacteremia. Crit Care Med 40: 1157-1163. doi:10.1097/CCM.0b013e3182377906. PubMed: 22080633. 22080633 PMC3288436

[40] Le Berre R, Nguyen S, Nowak E, Kipnis E, Pierre M et al. (2011) Relative contribution of three main virulence factors in *Pseudomonas* *aeruginosa* pneumonia. Crit Care Med 39: 2113-2120. doi:10.1097/CCM.0b013e31821e899f. PubMed: 21572326. 21572326

[41] Sayner SL, Frank DW, King J, Chen H, VandeWaa J et al. (2004) Paradoxical cAMP-induced lung endothelial hyperpermeability revealed by *Pseudomonas* *aeruginosa* ExoY. Circ Res 95: 196-203. doi:10.1161/01.RES.0000134922.25721.d9. PubMed: 15192021. 15192021

[42] Suttorp N, Hessz T, Seeger W, Wilke A, Koob R et al. (1988) Bacterial exotoxins and endothelial permeability for water and albumin *in* *vitro* . Am J Physiol 255: C368-C376. PubMed: 3138913. 3138913 10.1152/ajpcell.1988.255.3.C368

[43] Engel J, Balachandran P (2009) Role of *Pseudomonas* *aeruginosa* type III effectors in disease. Curr Opin Microbiol 12: 61-66. doi:10.1016/j.mib.2008.12.007. PubMed: 19168385. 19168385

[44] Sato H, Frank DW, Hillard CJ, Feix JB, Pankhaniya RR et al. (2003) The mechanism of action of the *Pseudomonas* *aeruginosa*-encoded type III cytotoxin, ExoU. EMBO J 22: 2959-2969. doi:10.1093/emboj/cdg290. PubMed: 12805211. 12805211 PMC162142

[45] Sato H, Frank DW (2004) ExoU is a potent intracellular phospholipase. Mol Microbiol 53: 1279-1290. doi:10.1111/j.1365-2958.2004.04194.x. PubMed: 15387809. 15387809

[46] Anderson DM, Schmalzer KM, Sato H, Casey M, Terhune SS et al. (2011) Ubiquitin and ubiquitin-modified proteins activate the *Pseudomonas* *aeruginosa* T3SS cytotoxin, ExoU. Mol Microbiol 82: 1454-1467. doi:10.1111/j.1365-2958.2011.07904.x. PubMed: 22040088. 22040088 PMC3237844

[47] Anderson DM, Frank DW (2012) Five mechanisms of manipulation by bacterial effectors: a ubiquitous theme. PLoS Pathog 8: e1002823 PubMed: 22927812. 22927812 10.1371/journal.ppat.1002823 PMC3426537

[48] Housley NA, Winkler HH, Audia JP (2011) The *Rickettsia* *prowazekii* ExoU homologue possesses phospholipase A_1_ (PLA_1_), PLA_2_, and lyso-PLA_2_ activities and can function in the absence of any eukaryotic cofactors *in* *vitro* . J Bacteriol 193: 4634-4642. doi:10.1128/JB.00141-11. PubMed: 21764940. 21764940 PMC3165714

[49] Sato H, Feix JB, Frank DW (2006) Identification of superoxide dismutase as a cofactor for the *Pseudomonas* type III toxin, ExoU. Biochemistry 45: 10368-10375. doi:10.1021/bi060788j. PubMed: 16922513. 16922513

[50] Barbieri JT, Sun J (2004) *Pseudomonas* *aeruginosa* ExoS and ExoT. Rev Physiol Biochem Pharmacol 152: 79-92. PubMed: 15375697. 15375697 10.1007/s10254-004-0031-7

[51] Frank DW (1997) The exoenzyme S regulon of *Pseudomonas* *aeruginosa* . Mol Microbiol 26: 621-629. doi:10.1046/j.1365-2958.1997.6251991.x. PubMed: 9427393. 9427393

[52] Shaver CM, Hauser AR (2004) Relative contributions of Pseudomonas aeruginosa ExoU, ExoS, and ExoT to virulence in the lung. Infect Immun 72: 6969-6977. 15557619 10.1128/IAI.72.12.6969-6977.2004 PMC529154

[53] Diaz MH, Shaver CM, King JD, Musunuri S, Kazzaz JA et al. (2008) *Pseudomonas* *aeruginosa* induces localized immunosuppression during pneumonia. Infect Immun 76: 4414-4421. doi:10.1128/IAI.00012-08. PubMed: 18663007. 18663007 PMC2546837

[54] Galán JE, Wolf-Watz H (2006) Protein delivery into eukaryotic cells by type III secretion machines. Nature 444: 567-573. doi:10.1038/nature05272. PubMed: 17136086. 17136086

[55] Sato H, Frank DW (2011) Multi-Functional Characteristics of the *Pseudomonas* *aeruginosa* Type III Needle-Tip Protein, PcrV; Comparison to Orthologs in other Gram-negative Bacteria. Front Microbiol 2: 142 PubMed: 21772833. 21772833 10.3389/fmicb.2011.00142 PMC3131520

[56] Sato H, Hunt ML, Weiner JJ, Hansen AT, Frank DW (2011) Modified needle-tip PcrV proteins reveal distinct phenotypes relevant to the control of type III secretion and intoxication by *Pseudomonas* *aeruginosa* . PLOS ONE 6: e18356. doi:10.1371/journal.pone.0018356. PubMed: 21479247. 21479247 PMC3066235

[57] Allmond LR, Karaca TJ, Nguyen VN, Nguyen T, Wiener-Kronish JP et al. (2003) Protein binding between PcrG-PcrV and PcrH-PopB/PopD encoded by the pcrGVH-popBD operon of the *Pseudomonas* *aeruginosa* type III secretion system. Infect Immun 71: 2230-2233. doi:10.1128/IAI.71.4.2230-2233.2003. PubMed: 12654846. 12654846 PMC152033

[58] Goure J, Pastor A, Faudry E, Chabert J, Dessen A et al. (2004) The V antigen of *Pseudomonas* *aeruginosa* is required for assembly of the functional PopB/PopD translocation pore in host cell membranes. Infect Immun 72: 4741-4750. doi:10.1128/IAI.72.8.4741-4750.2004. PubMed: 15271936. 15271936 PMC470589

[59] Blocker A, Gounon P, Larquet E, Niebuhr K, Cabiaux V et al. (1999) The tripartite type III secreton of *Shigella* *flexneri* inserts IpaB and IpaC into host membranes. J Cell Biol 147: 683-693. doi:10.1083/jcb.147.3.683. PubMed: 10545510. 10545510 PMC2151192

[60] Goure J, Broz P, Attree O, Cornelis GR, Attree I (2005) Protective anti-V antibodies inhibit *Pseudomonas* and *Yersinia* translocon assembly within host membranes. J Infect Dis 192: 218-225. doi:10.1086/430932. PubMed: 15962216. 15962216

[61] Neyt C, Cornelis GR (1999) Insertion of a Yop translocation pore into the macrophage plasma membrane by *Yersinia* *enterocolitica*: requirement for translocators YopB and YopD, but not LcrG. Mol Microbiol 33: 971-981. doi:10.1046/j.1365-2958.1999.01537.x. PubMed: 10476031. 10476031

[62] Neely AN, Holder IA, Wiener-Kronish JP, Sawa T (2005) Passive anti-PcrV treatment protects burned mice against *Pseudomonas* *aeruginosa* challenge. Burns 31: 153-158. doi:10.1016/j.burns.2004.09.002. PubMed: 15683685. 15683685

[63] Sawa T, Yahr TL, Ohara M, Kurahashi K, Gropper MA et al. (1999) Active and passive immunization with the *Pseudomonas* V antigen protects against type III intoxication and lung injury. Nat Med 5: 392-398. doi:10.1038/7391. PubMed: 10202927. 10202927

[64] Alvarez DF, King JA, Weber D, Addison E, Liedtke W et al. (2006) Transient receptor potential vanilloid 4-mediated disruption of the alveolar septal barrier: a novel mechanism of acute lung injury. Circ Res 99: 988-995. doi:10.1161/01.RES.0000247065.11756.19. PubMed: 17008604. 17008604 PMC2562953

[65] King J, Hamil T, Creighton J, Wu S, Bhat P et al. (2004) Structural and functional characteristics of lung macro- and microvascular endothelial cell phenotypes. Microvasc Res 67: 139-151. doi:10.1016/j.mvr.2003.11.006. PubMed: 15020205. 15020205

[66] Parker JC, Stevens T, Randall J, Weber DS, King JA (2006) Hydraulic conductance of pulmonary microvascular and macrovascular endothelial cell monolayers. Am J Physiol Lung Cell Mol Physiol 291: L30-L37. doi:10.1152/ajplung.00317.2005. PubMed: 16760315. 16760315

[67] Birukov KG, Birukova AA, Dudek SM, Verin AD, Crow MT et al. (2002) Shear stress-mediated cytoskeletal remodeling and cortactin translocation in pulmonary endothelial cells. Am J Respir Cell Mol Biol 26: 453-464. doi:10.1165/ajrcmb.26.4.4725. PubMed: 11919082. 11919082

[68] Birukova AA, Tian Y, Meliton A, Leff A, Wu T et al. (2012) Stimulation of Rho signaling by pathologic mechanical stretch is a "second hit" to Rho-independent lung injury induced by IL-6. Am J Physiol Lung Cell Mol Physiol 302: L965-L975. doi:10.1152/ajplung.00292.2011. PubMed: 22345573. 22345573 PMC3362159

[69] Dacheux D, Goure J, Chabert J, Usson Y, Attree I (2001) Pore-forming activity of type III system-secreted proteins leads to oncosis of *Pseudomonas* *aeruginosa*-infected macrophages. Mol Microbiol 40: 76-85. doi:10.1046/j.1365-2958.2001.02368.x. PubMed: 11298277. 11298277

[70] Broz P, Monack DM (2011) Molecular mechanisms of inflammasome activation during microbial infections. Immunol Rev 243: 174-190. doi:10.1111/j.1600-065X.2011.01041.x. PubMed: 21884176. 21884176 PMC3170129

[71] Ogura Y, Sutterwala FS, Flavell RA (2006) The inflammasome: first line of the immune response to cell stress. Cell 126: 659-662. doi:10.1016/j.cell.2006.08.002. PubMed: 16923387. 16923387

[72] Saleh M, Green DR (2007) Caspase-1 inflammasomes: choosing between death and taxis. Cell Death Differ 14: 1559-1560. doi:10.1038/sj.cdd.4402203. PubMed: 17703235. 17703235

[73] Zhao Y, Yang J, Shi J, Gong YN, Lu Q et al. (2011) The NLRC4 inflammasome receptors for bacterial flagellin and type III secretion apparatus. Nature 477: 596-600. doi:10.1038/nature10510. PubMed: 21918512. 21918512

[74] Miao EA, Ernst RK, Dors M, Mao DP, Aderem A (2008) *Pseudomonas* *aeruginosa* activates caspase 1 through Ipaf. Proc Natl Acad Sci U S A 105: 2562-2567. doi:10.1073/pnas.0712183105. PubMed: 18256184. 18256184 PMC2268176

[75] Vallis AJ, Finck-Barbançon V, Yahr TL, Frank DW (1999) Biological effects of *Pseudomonas* *aeruginosa* type III-secreted proteins on CHO cells. Infect Immun 67: 2040-2044. PubMed: 10085057. 10085057 10.1128/iai.67.4.2040-2044.1999 PMC96567

[76] Chatterjee A, Dimitropoulou C, Drakopanayiotakis F, Antonova G, Snead C et al. (2007) Heat shock protein 90 inhibitors prolong survival, attenuate inflammation, and reduce lung injury in murine sepsis. Am J Respir Crit Care Med 176: 667-675. doi:10.1164/rccm.200702-291OC. PubMed: 17615388. 17615388 PMC1994236

[77] Troyanovsky B, Alvarez DF, King JA, Schaphorst KL (2008) Thrombin enhances the barrier function of rat microvascular endothelium in a PAR-1-dependent manner. Am J Physiol Lung Cell Mol Physiol 294: L266-L275. PubMed: 18083763. 18083763 10.1152/ajplung.00107.2007

[78] Leavesley SJ, Annamdevula N, Boni J, Stocker S, Grant K et al. (2012) Hyperspectral imaging microscopy for identification and quantitative analysis of fluorescently-labeled cells in highly autofluorescent tissue. J Biophotonics 5: 67-84. doi:10.1002/jbio.201100066. PubMed: 21987373. 21987373 PMC3517021

