# Supplementary figures and images for: In the Absence of Effector Proteins, the Pseudomonas aeruginosa Type Three Secretion System Needle Tip Complex Contributes to Lung Injury and Systemic Inflammatory Responses
Source: PLoS One. 2013 Nov 27;8(11):e81792. doi: 10.1371/journal.pone.0081792 (PMC3842252; doi:10.1371/journal.pone.0081792)

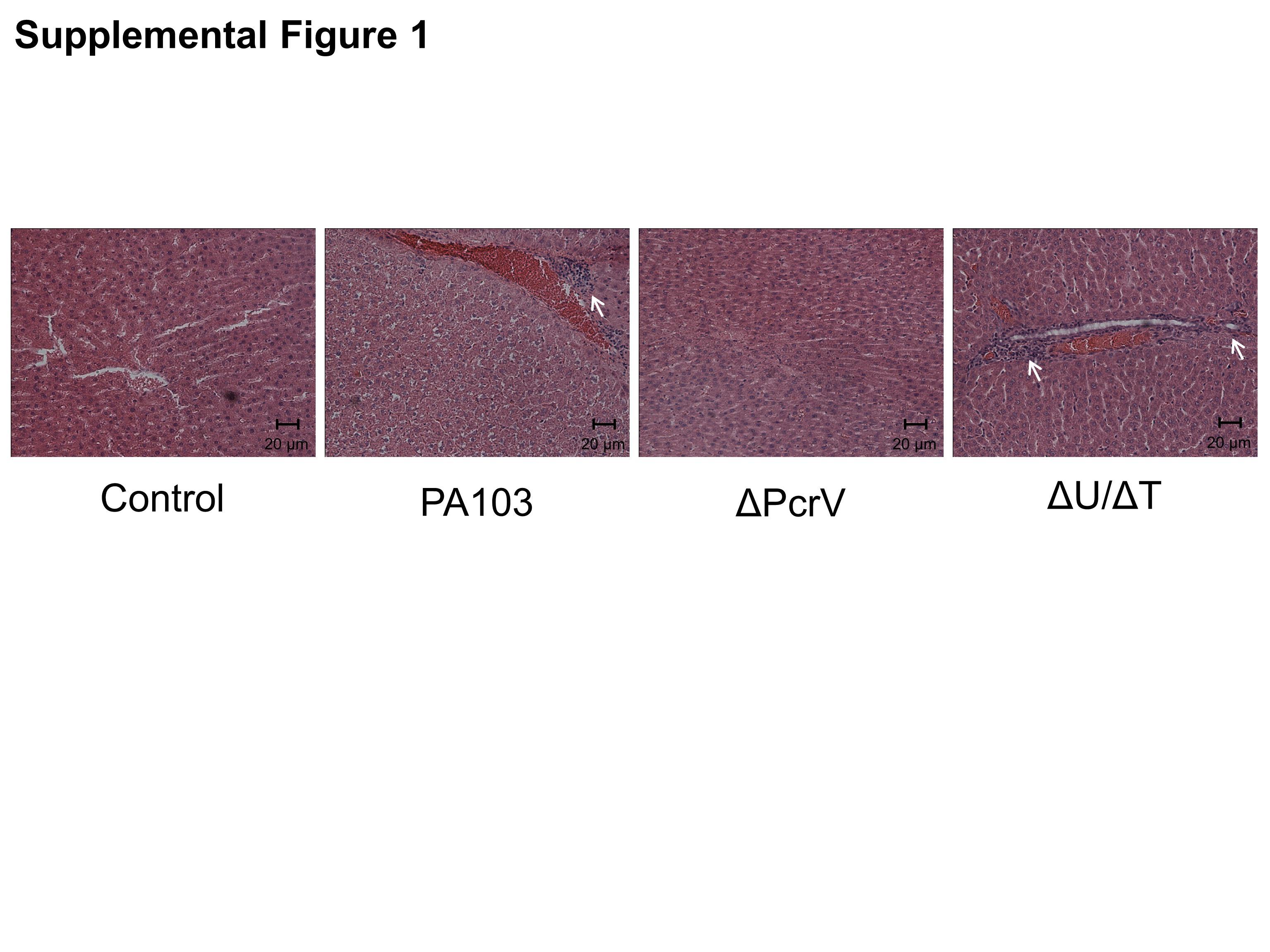

Supplement: Figure S1 — P. aeruginosa infection causes liver injury in a rat model of lung infection. Histological hematoxylin and eosin image of control liver and from animals inoculated with PA103, PA103 (ΔPcrV), and with PA103 (ΔU/ΔT). Arrows point to regions with significant peri-portal inflammation only in animals inoculated with the wild type PA103 and PA103 (ΔU/ΔT) indicating that the presence of a functional T3SS needle tip complex is sufficient to elicit injury. These data are consistent with the observed elevated biomarkers of liver injury (see Table 1 ). (TIF) [file pone.0081792.s001.tif]

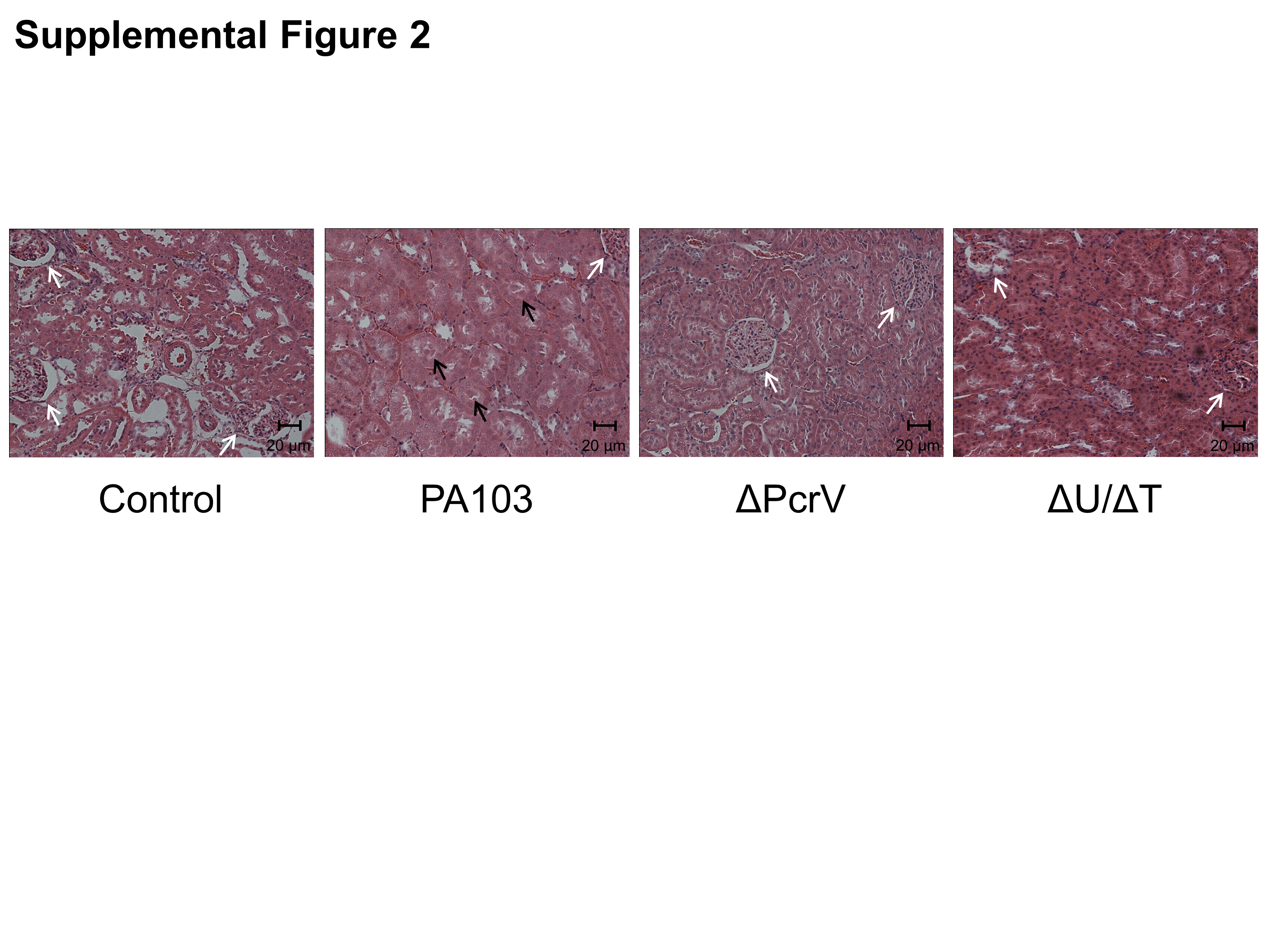

Supplement: Figure S2 — P. aeruginosa infection causes kidney injury in a rat model of lung infection. Histological hematoxylin and eosin image of control kidney and from animals inoculated with PA103, PA103 (ΔPcrV), and with PA103 (ΔU/ΔT). White arrows point to glomeruli as a point of reference. Black arrows point to tubular cast indicative of sepsis-induced injury that was observed only in animals inoculated with the wild type PA103. (TIF) [file pone.0081792.s002.tif]

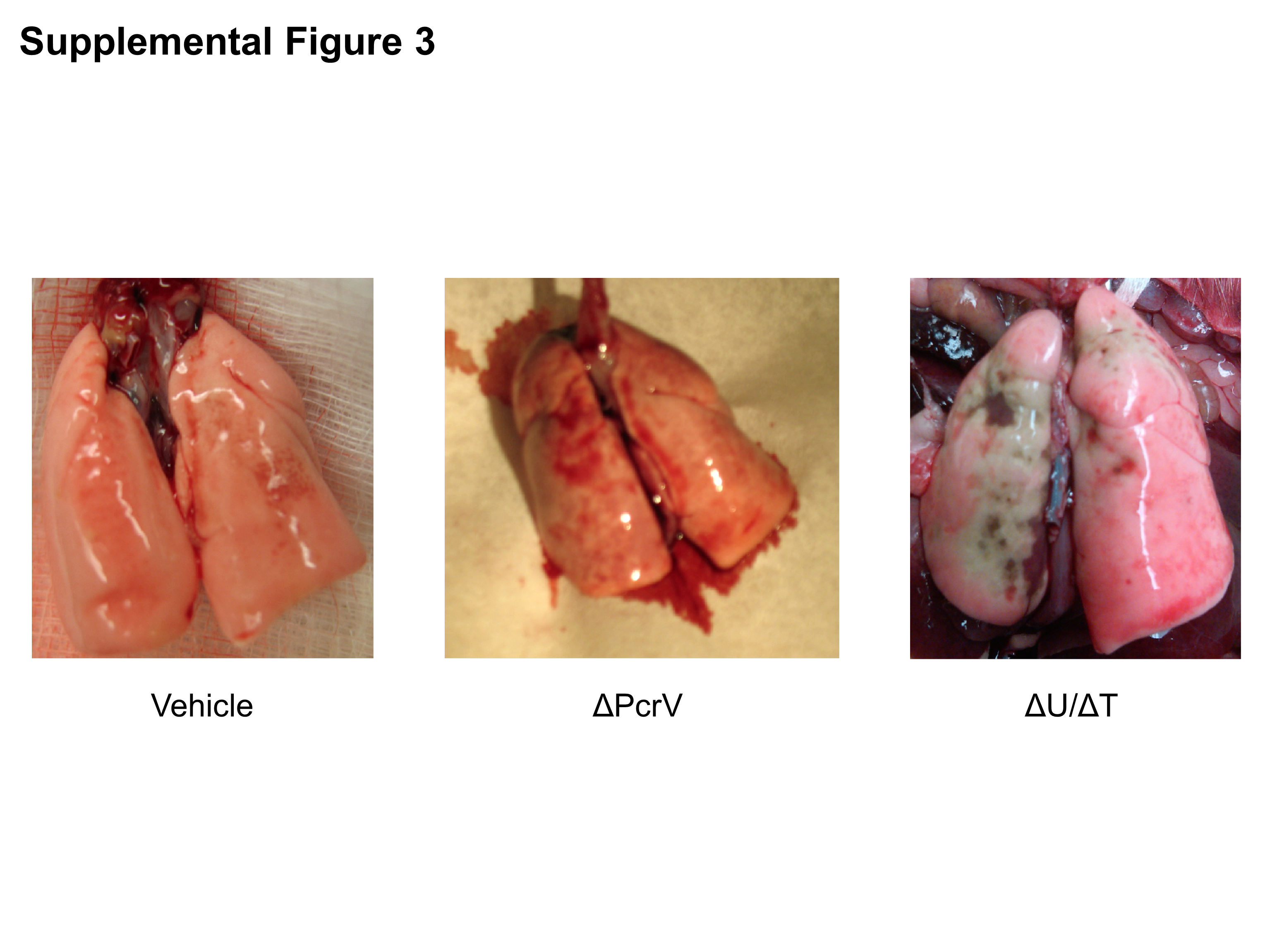

Supplement: Figure S3 — P. aeruginosa T3SS needle tip complex protein PcrV associates with lung injury. Macroscopic images of lungs at 48-hours post-inoculation. The left panel shows digital images of lungs from an animal inoculated with saline solution (vehicle control), the middle panel from an animal inoculated with ~1 x 10^7^ CFUs of PA103 (ΔPcrV), and the right panel from an animal inoculated with ~1 x 10^7^ CFUs of PA103 (ΔU/ΔT). PA103 (ΔU/ΔT) caused more gross damage to the lung compared to PA103 (ΔPcrV). (TIF) [file pone.0081792.s003.tif]

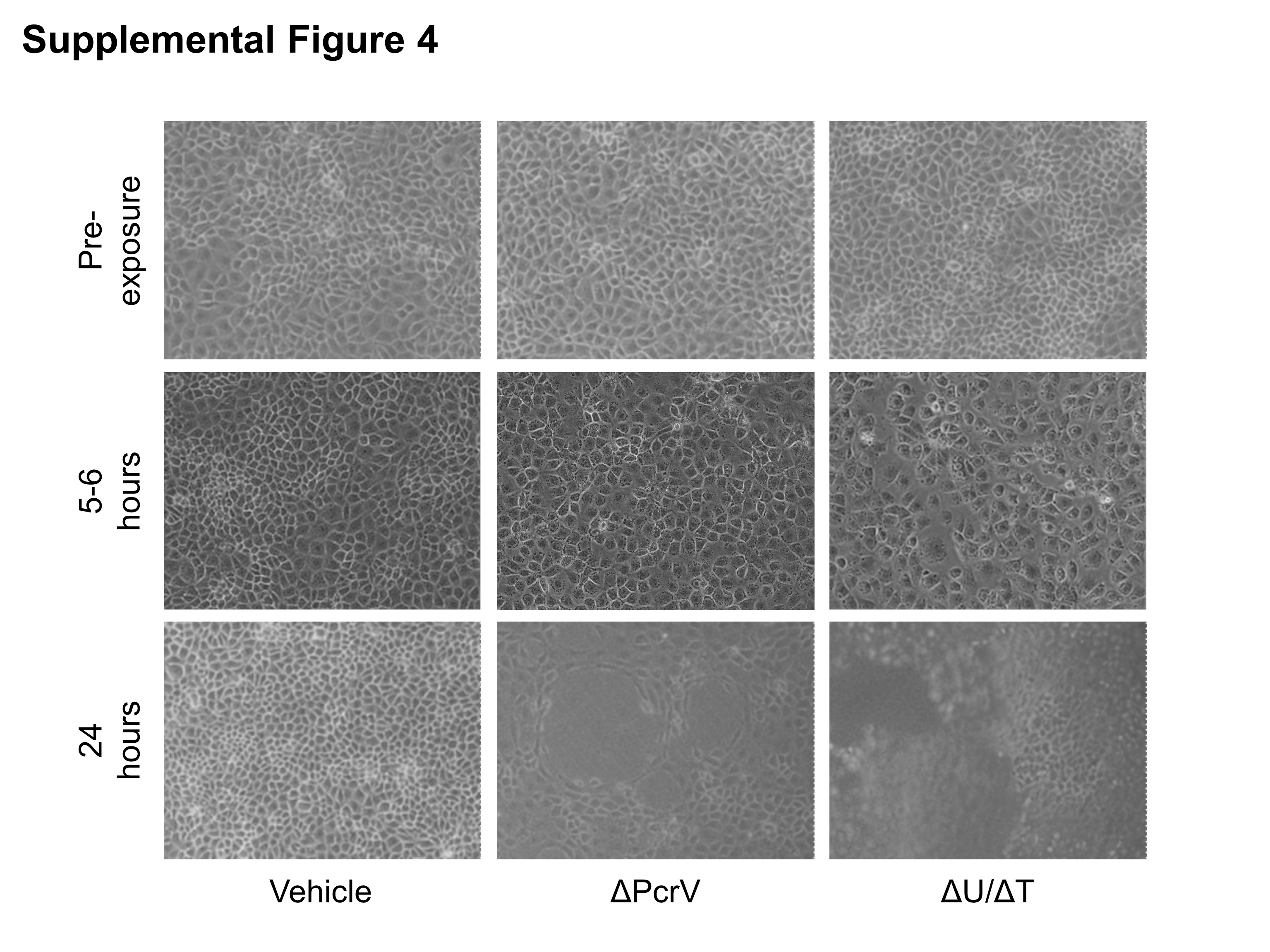

Supplement: Figure S4 — P. aeruginosa T3SS needle tip complex protein PcrV associates with PMVEC barrier disruption. Isolated rat PMVECs were grown in culture dishes to confluence and monolayer integrity was assessed by time-lapsed light microscopy. PMVECs were inoculated with saline solution (Vehicle control), or with either P. aeruginosa mutant at a 40:1 MOI. Exposures of the same region of the culture dish were obtained pre-exposure, at 5-6-hours, and at 24-hours post-inoculation. PMVECs inoculated with PA103 (ΔU/ΔT) begin to alter morphology during the early phase of infection (5-hours post-inoculation) compared to PMVECs inoculated with PA103 (ΔPcrV) and uninoculated PMVEC controls. During the late phase of infection PA103 (ΔU/ΔT) caused more PMVEC damage and loss of cell-to-cell adhesion compared to inoculation with PA103 (ΔPcrV) and uninoculated PMVEC controls. The PA103 (ΔPcrV) mutant also caused barrier disruption, but in a protracted fashion. Representative images are shown. Experiments were repeated at least 10 times per condition. (TIF) [file pone.0081792.s004.tif]

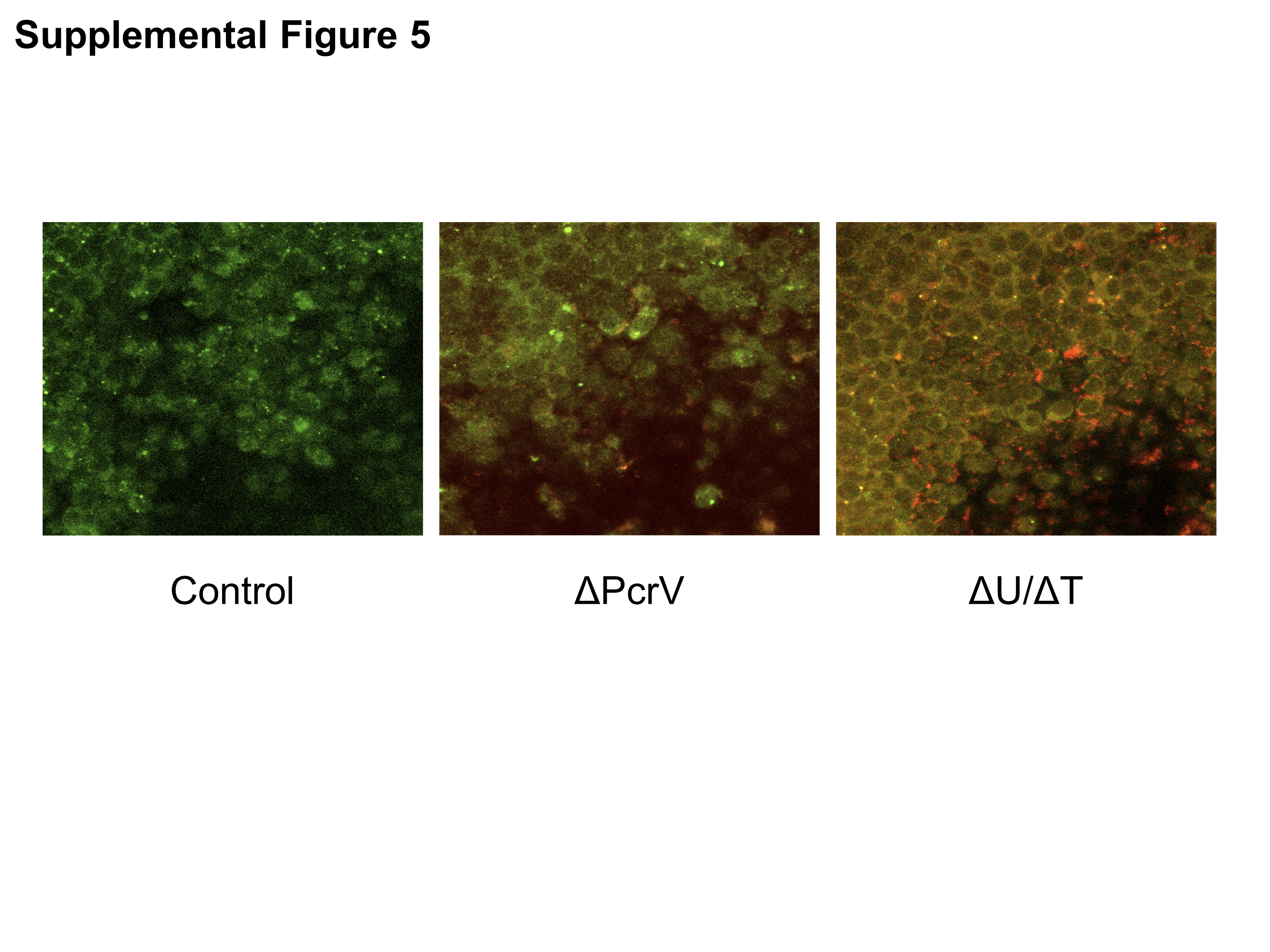

Supplement: Figure S5 — P. aeruginosa T3SS needle tip complex protein PcrV associates with PMVEC cell death during the late phase of infection. Isolated rat PMVECs were grown on glass cover slips to confluence and cell death was assessed by TUNEL staining and visualized via confocal fluorescence microscopy and hyperspectral imaging. PMVECs were inoculated with saline solution (vehicle control), or with either P. aeruginosa mutant at a 40:1 MOI. Slides were prepared for staining at 24-hours post-inoculation. PMVECs were stained with antibody against PECAM-1 (CD31, green pseudocolor) and TUNEL positive cells shown in red pseudocolor. PMVECs inoculated with PA103 (ΔU/ΔT) display significant number of TUNEL-positive cells compared to PMVECs inoculated with PA103 (ΔPcrV) and uninoculated PMVEC controls. (TIF) [file pone.0081792.s005.tif]
